# CD24 induced cellular quiescence-like state and chemoresistance in ovarian cancer cells via miR-130a/301a-dependent CDK19 downregulation

**DOI:** 10.1038/s41420-024-01858-y

**Published:** 2024-02-15

**Authors:** Yeonsue Jang, Suki Kang, Hyun Ho Han, Baek Gil Kim, Nam Hoon Cho

**Affiliations:** 1 Department of Pathology, Yonsei University College of Medicine, Seoul, Republic of Korea; 2 Brain Korea 21 Plus Project for Medical Science, Yonsei University College of Medicine, Seoul, Republic of Korea; 3 Department of Urology, Urological Science Institute, Yonsei University College of Medicine, Seoul, Republic of Korea; 4 Severance Biomedical Science Institute (SBSI), Yonsei University College of Medicine, Seoul, Republic of Korea

**Keywords:** Cancer stem cells, Ovarian cancer, Cell signalling

## Abstract

Cancer stem-like cell (CSC) is thought to be responsible for ovarian cancer recurrence. CD24 serves as a CSC marker for ovarian cancer and regulates the expression of miRNAs, which are regulators of CSC phenotypes. Therefore, CD24-regulated miRNAs may play roles in manifesting the CSC phenotypes in ovarian cancer cells. Our miRNA transcriptome analysis showed that 94 miRNAs were up or down-regulated in a CD24-high clone from an ovarian cancer patient compared to a CD24-low one. The CD24-dependent expression trend of the top 7 upregulated miRNAs (miR-199a-3p, 34c, 199a-5p, 130a, 301a, 214, 34b*) was confirmed in other 8 clones (4 clones for each group). CD24 overexpression upregulated the expression of miR-199a-3p, 34c, 199a-5p, 130a, 301a, 214, and 34b* in TOV112D (CD24-low) cells compared to the control, while CD24 knockdown downregulated the expression of miR-199a-3p, 199a-5p, 130a, 301a, and 34b* in OV90 (CD24-high) cells. miR-130a and 301a targeted CDK19, which induced a cellular quiescence-like state (increased G0/G1 phase cell population, decreased cell proliferation, decreased colony formation, and decreased RNA synthesis) and resistance to platinum-based chemotherapeutic agents. CD24 regulated the expression of miR-130a and 301a via STAT4 and YY1 phosphorylation mediated by Src and FAK. miR-130a and 301a were positively correlated in expression with CD24 in ovarian cancer patient tissues and negatively correlated with CDK19. Our results showed that CD24 expression may induce a cellular quiescence-like state and resistance to platinum-based chemotherapeutic agents in ovarian cancer via miR-130a and 301a upregulation. CD24-miR-130a/301a-CDK19 signaling axis could be a prognostic marker for or a potential therapeutic target against ovarian cancer recurrence.

## Introduction

CD24 is a surface receptor linked to downstream networks, a cancer stem-like cell (CSC) marker for ovarian cancer [1], and induces miRNA expression. Therefore, the phenotypic manifestation of ovarian CSCs may be associated with CD24-associated expression of miRNAs. Ovarian cancer frequently recurs after chemotherapy, the current standard care for ovarian cancer. A significant cause of post-treatment recurrence is the presence of CSCs. Therefore, CD24-associated expression of miRNAs and their regulated pathways can be therapeutic targets for ovarian CSCs. However, the relation between CD24 and miRNA expression and its roles in CSC phenotype acquisition in ovarian cancer remains unknown.

CD24 expression is associated with CSC phenotype acquisition in various cancers. In liver cancer, CD24 expression was associated with self-renewal and cancer initiation [2]. In pancreatic cancer, a highly tumorigenic subpopulation of cancer cells expressed CD24 [3]. In colorectal cancer, CD24 expression was associated with tumor initiation, self-renewal, and differentiation ability to multiple lineages [4]. In ovarian cancer, the CD24-positive population of cancer cells showed significantly greater tumor initiation in a transgenic murine model than the CD24-negative population [5]. CD24 overexpression induced epithelial-to-mesenchymal transition (EMT), one of the biological processes generating CSCs [6], and resistance to cisplatin in Caov-3 cells [6]. CD24-positive population enriched from an ovarian cancer patient showed stemness-related gene overexpression, resistance to cisplatin, and tumor initiation [1].

CD24 can contribute to cancer progression as a surface receptor associated with various downstream networks. CD24 alters Signal Transducer And Activator Of Transcription 3 (STAT3) expression via SRC Proto-Oncogene, Non-Receptor Tyrosine Kinase (Src) [7], which leads to tumor invasion and metastasis [8]. CD24-dependent activation of Src is associated with the downregulation of tissue factor pathway inhibitor 2 (TFPI-2), a tumor suppressor gene [9]. CD24 overexpression induces mutational and viral oncogene-mediated p53 inactivation in prostate cancer by disrupting ARF-NPM interaction [10]. The genetic ablation and therapeutic blockade of CD24 resulted in a macrophage-dependent reduction of tumor growth and extension of survival in vivo ovarian and breast cancer models [11].

CD24 may play a role in CSC phenotype manifestation by regulating miRNA expression. It was reported that CD24 induces miR-21 expression via Src activation [12]. Accumulated evidence showed that miRNAs regulate CSCs. let-7 miRNA reduced breast CSC properties in vitro and in a mouse model [13]. miR-200c modulated the expression of BMI1 Proto-Oncogene, Polycomb Ring Finger (BMI1), a regulator of stem cell self-renewal, and inhibited the clonal expansion of breast cancer cells [14]. miR-22 enhanced stem cell function and induced hematological transformation by targeting Tet Methylcytosine Dioxygenase 2 (TET2) [15]. miR-199b-5p impaired CSCs in medulloblastoma by regulating Hes Family BHLH Transcription Factor 1 (HES1) [16]. In ovarian cancer, miR-328-3p was significantly upregulated, and its inhibition impaired CSC function and metastasis [17]. miR-1207 overexpression promoted CSC features by activating the Wnt/β-catenin signaling pathway [18]. miR-136 inhibited CSC activity and enhanced the resistance to paclitaxel by targeting Notch Receptor 3 (Notch3) [19].

In this study, we mined the miRNAs depending on CD24 expression from primary ovarian cancer cells. We demonstrated that some of the miRNAs were associated with CSC phenotypes and can be therapeutic targets for ovarian cancer stem cells.

## Results

### CD24 expression affected miRNA transcriptome in ovarian cancer cells

**Fig. 1:**
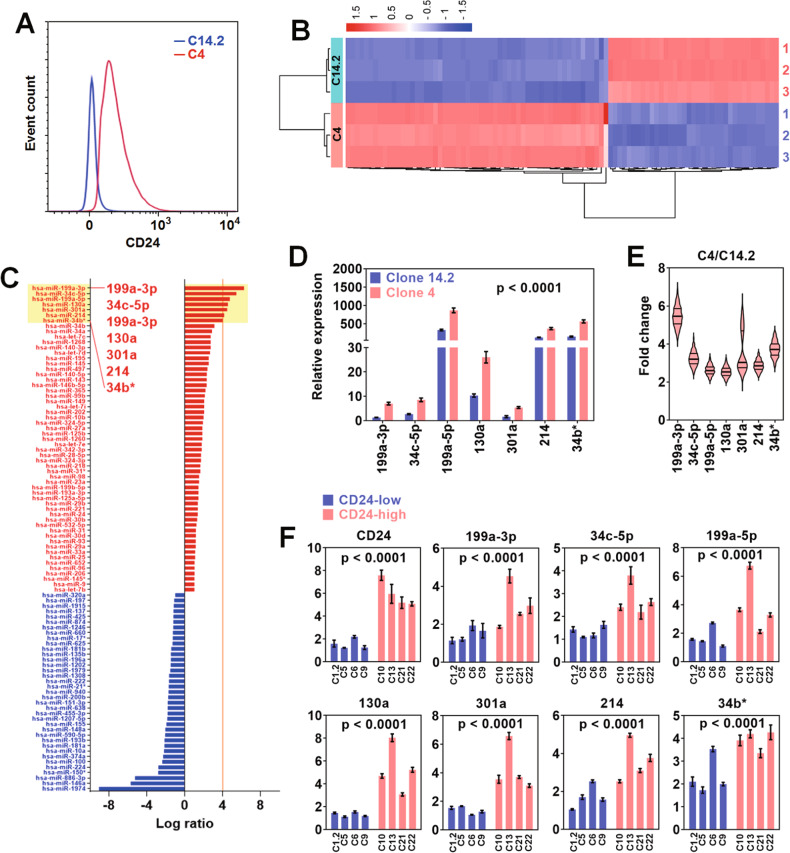
The comparative analysis of CD24-dependent miR expression in ovarian cancer cells. **A** The flow cytometric analysis of CD24 expression in primary ovarian cancer cell clones (C14.2 and C4). The cell clones were isolated from an ovarian cancer patient. **B** The heatmap display of the miRNA array for C14.2 and C4 clones. The array for each clone was run in triplicate. **C** The bar graph presentation of the miRNAs up- or down-regulated in the C4 clone compared to the C14.2 clone. **D** The real-time PCR analysis, and **E** the fold change analysis of the 7 most upregulated miRNAs in the C4 clone compared to the C14.2 clone. Relative expression was calculated using the ΔΔCt method. **F** The expression comparison analysis of the 7 most upregulated miRNAs between CD24-low and high clones. All the clones were derived from the same ovarian cancer patient.

For miRNA transcriptome analysis, two primary ovarian cell clones with CD24-low (CD14.2) and CD24-high (C4) expression were obtained from an ovarian cancer patient (Fig. 1A). 159 miRNAs were differentially expressed between C14.2 and C4 (Fig. 1B). In Fig. 1C, among the miRNAs, 94 miRNAs (57 upregulated, 37 downregulated) showed a more than 2-fold difference between C14.2 and C2. In particular, miR-199a-3p, 34c, 199a-5p, 130a, 301a, 214, and 34b* were upregulated above 15-fold in C4 compared to C14.2. In real-time PCR analysis, the seven upregulated miRNAs were significantly upregulated in C4 compared to C14.2 (Fig. 1D), and their fold changes (C4/C14.2) distributed ~2.5 folds to 6 folds (Fig. 1E). The 7 upregulated miRNAs were confirmed in other primary ovarian cell clones obtained from the same ovarian cancer patient. In Fig. 1F, the other clones also demonstrated a similar expression pattern of the 7 miRNAs. CD24-high clones (C1.2, C5, C6, and C9) showed a significantly higher expression of the 7 miRNAs than C24-low clones (C10, C13, C21, and C22).

### CD24 overexpression induced cancer stem-like cell features and regulated the expression of miRNAs in ovarian cancer cells

**Fig. 2:**
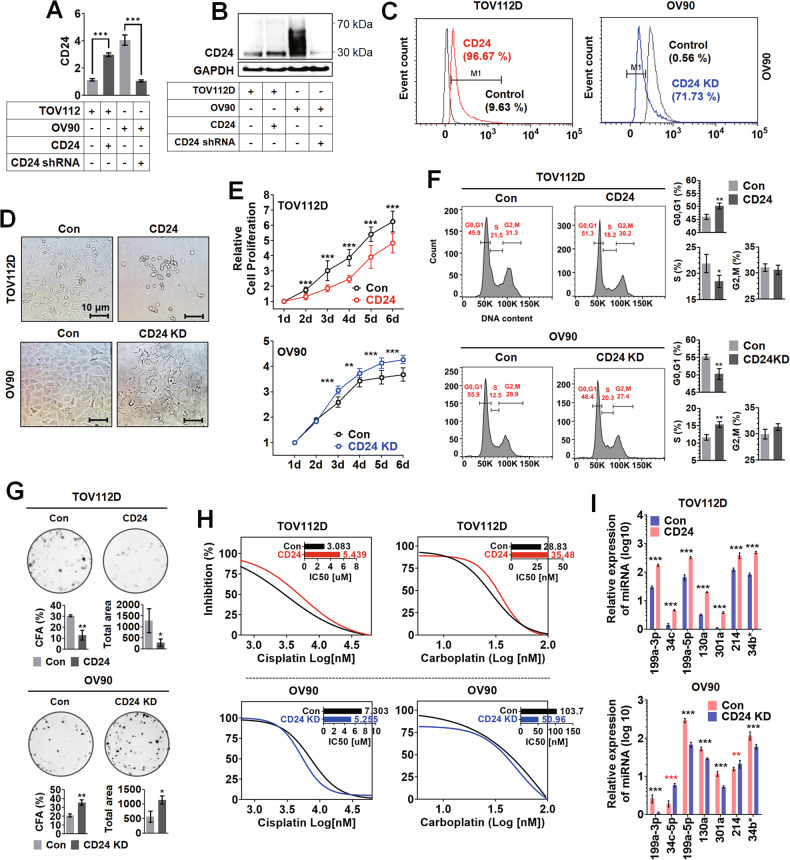
CD24 expression-dependent alterations in ovarian cancer cells. **A** Real-time PCR analysis, **B** Western blot analysis, and **C** flow cytometric analysis of CD24 expression in CD24 expression-manipulated ovarian cancer cells. Comparative analyses of **D** morphology, **E** cell proliferation, **F** cell cycle, **G** colony forming ability, **H** IC50, and **I** CD24-regulated miRNA expression of CD24 expression-manipulated ovarian cancer cells.

CD24 expression was manipulated in ovarian cancer cell lines, TOV112D (CD24-low) and OV90 (CD24-high) cells, using gene transduction to confirm CD24-dependent upregulation of miR-199-3p, 34c, 199a-5p, 130a, 301a, 214, and 34b*. The transcript level of CD24 was upregulated in CD24-overexpressing TOV112D cells compared to the parental TOV112D cells, while that was downregulated in CD24-knockdown OV90 cells compared to the parental OV90 cells (Fig. 2A). Like transcript expression levels, CD24 protein was increased in the CD24-overexpressing TOV112 cells compared to the parental TOV112 cells, whereas that was decreased in the CD24-knockdown OV90 cells compared to the parental OV90 cells (Fig. 2B), which was also confirmed by flow cytometry analysis (Fig. 2C). CD24 expression altered the morphology of TOV112D and OV90 cells. In Fig. 2D, CD24 overexpression induced the rounding of TOV112D cells, while CD24 knockdown changed the monolayer and epithelial shape to an irregularly piled one in OV90 cells. CD24 expression slowed the growth rate of ovarian cancer cells. CD24 overexpression significantly decreased the proliferation of TOV112D cells compared to the control, whereas CD24 knockdown increased the proliferation of OV90 cells compared to the control (Fig. 2E). Since CD24 expression was reported to be associated with CSC-like phenotypes in ovarian cancer, cell cycle, colony formation ability (CFA), and drug resistance were investigated. CD24 expression induced the cellular quiescence-like state in ovarian cancer cells. CD24 overexpression significantly increased the population of G0/G1 phase cells while decreasing that of S phase cells in TOV112 cells compared to the control. On the other hand, CD24 knockdown significantly reduced the population of G0/G1 phase cells while increasing that of S phase cells in OV90 cells compared to the control (Fig. 2F). CD24 expression reduced colony forming ability of ovarian cancer cells. CD24 overexpression significantly decreased the colony number and total area of TOV112D cells compared to the control, while CD24 knockdown increased those of OV90 cells compared to the control (Fig. 2G). CD24 expression increased the resistance of platinum-based chemotherapeutic agents. In treating cisplatin or carboplatin, the half-maximal inhibitory concentration (IC50) was increased (cisplatin: 3.083 to 5.439, carboplatin: 28.83 to 35.48) by CD24 overexpression in TOV112D cells compared to the control. On the other hand, IC50 was decreased (cisplatin: 7.303 to 5.255, carboplatin: 103.7 to 50.96) by CD24 knockdown in OV90 cells compared to the control (Fig. 2H). In Fig. 2I, similar to the results of primary ovarian cancer clones, CD24 overexpression induced a significant upregulation of miR-199a-3p, 34c, 199a-5p, 130a, 301a, 214, and 34b* in TOV112D cells. On the other hand, CD24 knockdown caused a significant downregulation of miR-199a-3p, 199a-5p, 130a, 301a, and 34b* in OV90 cells but not miR-34c and 214. CD24 was associated with the expression of stemness-related genes. In Supplementary Fig. S1A, CD24 overexpression significantly induced the upregulation of ATP Binding Cassette Subfamily G Member 2 (ABCG2), Aldehyde Dehydrogenase 1 Family Member A1 (ALDH1A1), BMI1, CD34, CD44, Cadherin 1 (CDH1), Catenin Alpha 1 (CTNNA1), Epithelial Cell Adhesion Molecule (EPCAM), HES1, KIT Proto-Oncogene, Receptor Tyrosine Kinase (KIT), Nestin (NES), Notch Receptor 1 (NOTCH1), Notch Receptor 4 (NOTCH4), POU Class 5 Homeobox 1 (POU5F1), Prominin 1 (PROM1), Smoothened, Frizzled Class Receptor (SMO), and Thy-1 Cell Surface Antigen (THY1) in TOV112D cells compared to the control. On the other hand, in Supplementary Fig. S1B, CD24 knockdown significantly induced the downregulation of ABCG2, EPCAM, NOTCH4, PROM1, and SMO in OV90 cells compared to the control, while the upregulation of BMI1, CD34, CD44, CTNNA1, NES, POU5F1, and THY1. According to putative target gene analysis using miRWalk, TargetScan, and TargetRank, there were no binding sites for the 7 miRNAs in these genes (data not shown). In the correlation analysis between the 7 miRNAs and stemness-related genes, miR-199a-3p, 199a-5p, 130a, 301a, and 34b* were positively and commonly correlated with ABCG2, PROM1, EPCAM, SMO, and NOTCH4 in TOV112D and OV90 cells (Supplementary Fig. S2).

### CD24-regulated miRNAs were associated with CSC phenotype manifestation

**Fig. 3:**
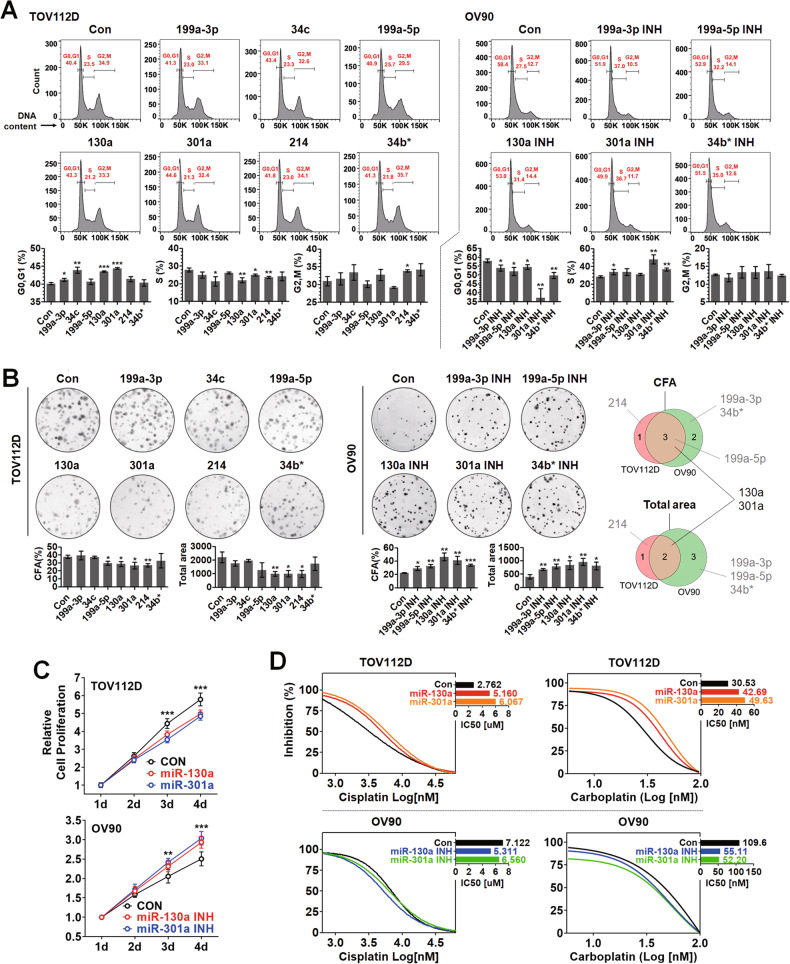
CSC-like phenotypes manifestation by CD24-regulated miRNA expression in ovarian cancer cells. Analyses of **A** cell cycle, **B** colony forming ability, **C** cell proliferation, and **D** IC50 in miRNA expression-manipulated ovarian cancer cells. TOV112D cells were transfected with miRNA plasmids. OV90 cells were transfected with miRNA inhibitor plasmids.

The miRNAs regulated by CD24 expression were named CD24-regulated miRNAs for convenience. The correlation between CD24 and CSC phenotypes led us to assume that CD24 may induce CSC phenotype manifestation in ovarian cancer cells via CD24-regulated miRNAs. In Fig. 3A, in TOV112D cells, compared to the control, the population of G0/G1 phase cells was significantly increased by the overexpression of miR-199a-3p, 34c, 130a, and 301a. The population of S phase cells was significantly decreased by the overexpression of miR-34c, 130a, 301a, and 214. The overexpression of miR-214 significantly increased the population of G2/M phase cells. On the other hand, in OV90 cells, compared to the control, the population of G0/G1 phase cells was significantly decreased by the inhibition of miR-199a-3p, 199a-5p, 130a, 301a, and 34b*. The population of S phase cells was increased by inhibiting miR-199a-3p, 301a, and 34b*. The transfection efficacy of overexpression and inhibitor (INH) plasmid vectors was evaluated by semi-quantitative PCR analysis (Supplementary Fig. S3). In Fig. 3B, the overexpression of miR-130a, 301a, and 214 significantly decreased the colony number and total area of TOV112D cells compared to the control. On the other hand, the inhibition of miR-199a-3p, 199a-5p, 130a, 301a, and 34b* significantly increased the colony number and total area of OV90 cells compared to the control. miR-130a and 301a commonly affected the colony number and total area of TOV112D and OV90 cells. Based on the cell cycle and CFA analyses, miR-130a and 301a were selected for further investigation. In Fig. 3C, the overexpression of miR-130a and 301a decreased the proliferation of TOV112D cells compared to the control. On the other hand, the inhibition of miR-130a and 301a increased the proliferation of OV90 cells compared to the control. In Fig. 3D, the overexpression of miR-130a and 301a increased IC50 values of cisplatin (2.762 to 5.160 for miR-130a and 6.067 for miR-301a) and carboplatin (30.53 to 42.69 for miR-130a and 49.63 for miR-301a) in TOV112D cells compared to the control. On the other hand, the inhibition of miR-130a and 301a decreased IC50 values of cisplatin (7.122 to 5.311 for miR-130a and 6.560 for miR-301a) and carboplatin (109.6 to 55.11 for miR-130a and 52.20 for miR-301a) in OV90 cells compared to the control.

### miR-130a and 301a induced CSC phenotype manifestation in ovarian cancer cells by targeting CDK19

**Fig. 4:**
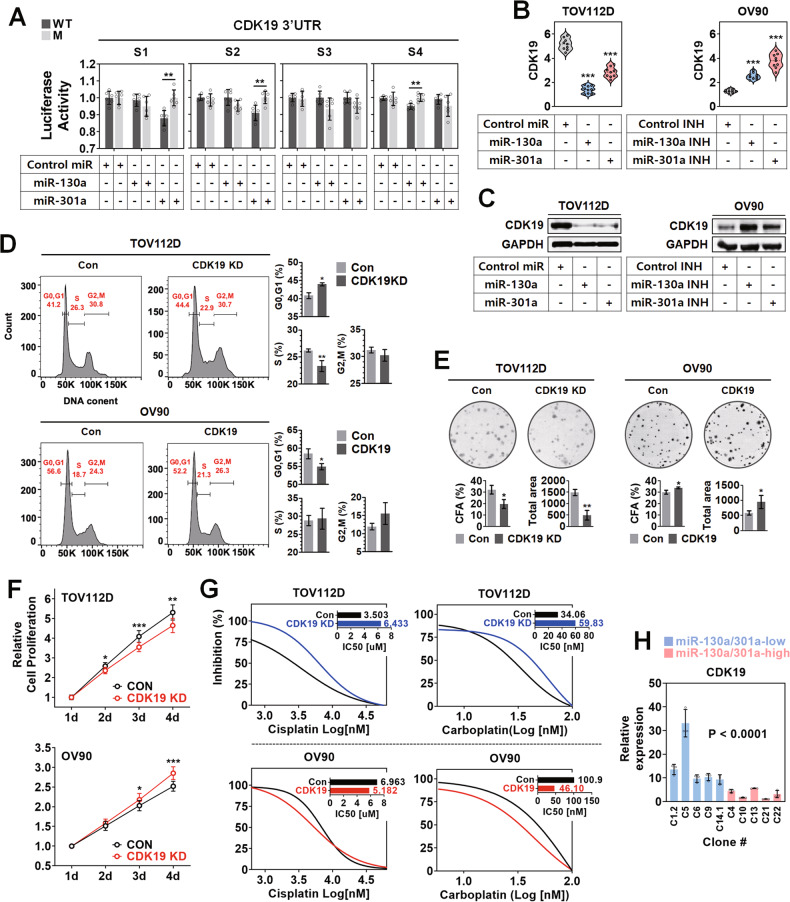
CSC-like phenotype manifestation by miR-130a and 301a-dependent CDK19 downregulation in ovarian cancer cells. **A** Binding assay of miR-130a and 301a to CDK19 3’UTR. **B** Real-time PCR analysis and **C** Western blot analysis of CDK19 expression in miR-130a or 301a expression-manipulated ovarian cancer cells. Analyses for **D** cell cycle, **E** Colony forming ability, **F** Cell proliferation, and **G** IC50 in CDK19-expression-manipulated ovarian cancer cells. **H** Expression analysis of CDK19 transcript in miR-130a/301a-low and high ovarian cancer clones. All the clones were derived from the same ovarian cancer patient.

According to miRNA database analysis (Supplementary Fig. S4), CDK19 was a common putative target gene of miR-130a and 301a, and its 3’ untranslated region (UTR) was predicted to have 4 sites for miR-130a and 301a. To validate the binding of miR-130a and 301a to CDK19 3’UTR, wild and seed sequence deletion-mutant CDK19 3’UTR plasmids were constructed, as shown in Supplementary Fig. S5. In Fig. 4A, the overexpression of miR-130a significantly decreased the luciferase activity of wild-type CDK19 3’UTR, while it did not that of site 4 (S4) deletion-mutant CDK19 3’UTR. The overexpression of miR-301a significantly decreased the luciferase activity of wild-type CDK19 3’UTR, whereas it did not that of site 1 (S1) and site 2 (S2) deletion-mutant CDK19 3’UTR. In Fig. 4B, the expression of CDK19 transcript was significantly decreased in TOV112D cells by the overexpression of miR-130a and 301a compared to the control. On the other hand, the expression of CDK19 transcript was significantly increased in OV90 cells by inhibiting miR-130a and 301a compared to the control. Like transcripts, CDK19 protein was significantly decreased in TOV112D cells by the overexpression of miR-130a and 301a compared to the control, while it was increased in OV90 cells by inhibiting miR-130a and 301a compared to the control (Fig. 4C). In Fig. 4D, CDK19 knockdown significantly increased the population of G0/G1 phase cells while decreasing that of S phase cells in TOV112D cells compared to the control. On the other hand, CDK19 overexpression significantly reduced the population of G0/G1 phase cells in OV90 cells compared to the control. In Fig. 4E, CDK19 knockdown significantly decreased the colony number and total area of TOV112D cells compared to the control. On the other hand, CDK19 overexpression significantly increased the colony number and total area of OV90 cells compared to the control. In Fig. 4F, CDK19 knockdown decreased the proliferation of TOV112D cells compared to the control, whereas CDK19 overexpression increased the proliferation of OV90 cells compared to the control. In Fig. 4G, CDK19 knockdown increased the IC50 values of cisplatin (from 3.503 to 6.433) and carboplatin (from 34.06 to 59.83) in TOV112D cells compared to the control, whereas CDK19 overexpression decreased the IC50 values of cisplatin (from 6.963 to 5.182) and carboplatin (from 100.9 to 46.10) in OV90 cells compared to the control. CDK19 expression was higher in miR-130a/301a-low clones than miR-130a/301a-high ones (Fig. 4H).

### STAT4 and YY1 regulated the expression of miR-130a and 301a

**Fig. 5:**
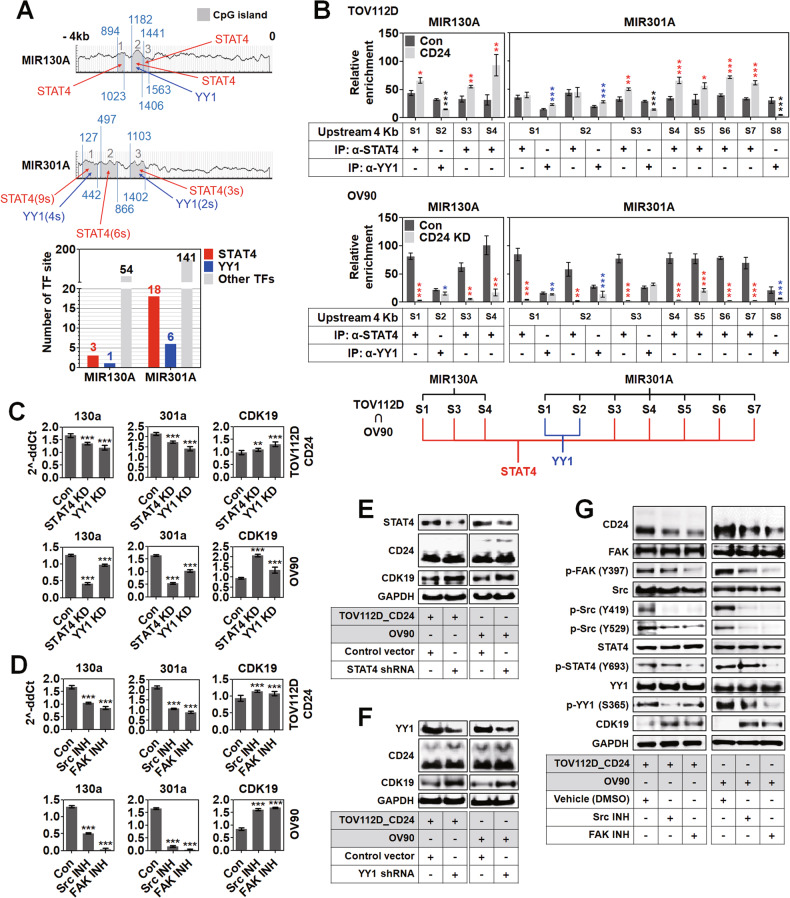
STAT4 and YY1-mediated regulation of miR-130a and 301a expression in CD24-high ovarian cancer cells. **A** Putative binding site for STAT4 and YY1 on MIR130A and MIR301A promoter regions. ※ s: site. **B** Chromatin immunoprecipitation of MIR130A and MIR301A promoter using anti-STAT4 and YY1 antibodies. ※ S: segment. Real-time PCR analysis of **C** STAT4 or YY1-dependent and **D** Src or FAK-mediated miR-130a, 301a, and CDK19 expression in CD24-high ovarian cancer cells. Western blot analysis of **E** STAT4-dependent, **F** YY1-dependent, and **G** Src or FAK-mediated CDK19 expression in CD24-high ovarian cancer cells.

Upstream 4 kb of MIR130A and MIR301A were analyzed to find putative binding transcription factors. As shown in Fig. 5A, 1 site for STAT4 on CpG island-1, 1 site for STAT4 and 1 site for YY1 on CpG island-2, and 1 site for STAT4 on CpG island-3 were found in the MIR130A promoter region. 9 sites for STAT4 and 4 sites for YY1 on CpG island-1, 6 sites for STAT4 on CpG island-2, and 3 sites for STAT4 and 2 sites for YY1 on CpG island-3 were found in the MIR301A promoter region. In total, 3 sites for STAT4 and 1 site for YY1 were predicted in the MIR130A 4 kb upstream region, while 18 sites for STAT4 and 6 sites for YY1 in the MIR301A 4 kb upstream region. Since some sites were located very closely, they were included in one segment for the convenience of ChIP assay. In Fig. 5B, the enrichment of segment (S)1, S3, and S4 on MIR130A and S3, S4, S5, S6, and S7 on MIR301A was significantly increased in CD24-overexpressing TOV112D cells by precipitation with anti-STAT4 antibody compared to the control. The enrichment of S1 and S2 on MIR301A was significantly increased by precipitation with anti-YY1 antibody compared to the control. On the other hand, the enrichment of S1, S3, and S4 on MIR130A and S1, S2, S3, S4, S5, S6, and S7 on MIR301A were significantly decreased in CD24-knockdown OV90 cells by precipitation with anti-STAT4 antibody compared to the control. The enrichment of S2 on MIR130A and S1, S2, and S8 on MIR301A was significantly decreased in CD24-knockdown OV90 cells by precipitation with anti-YY1 antibody compared to the control. According to our results, STAT4 commonly recognized S1, S3, S4 on MIR130A and S3, S4, S5, S6, and S7 on MIR301A in TOV112D and OV90 cells, while YY1 commonly recognized S1 and S2 on MIR301A in TOV112D and OV90 cells. In Fig. 5C, the transcript level of miR-130a and 301a was significantly decreased in CD24-overexpressing TOV112D and OV90 cells by the knockdown of STAT4 and YY1 compared to the control, while that of CDK19 was increased. In Fig. 5D, the transcript level of miR-130a and 301a was significantly decreased in CD24-overexpressing TOV112D and OV90 cells by treatment with Src or FAK inhibitor compared to the control, whereas that of CDK19 was increased. Like transcript levels, CDK19 protein was increased in CD24-overexpressing TOV112D and OV90 cells by the knockdown of STAT4 (Fig. 5E) or YY1 (Fig. 5F). In Fig. 5G, Src inhibitor treatment decreased the phosphorylation of FAK, Src, and YY1 in CD24-overexpressing TOV112D and OV90 cells, which increased the expression of CDK19 protein. On the other hand, FAK inhibitor treatment decreased the phosphorylation of FAK, Src, STAT4, and YY1 in CD24-overexpressing TOV112D and OV90 cells, which increased the expression of CDK19 protein. Src and FAK inhibitors also reduced the expression of CD24.

### CD24-miR-130a/301a-CDK19 signaling axis reduced RNA synthesis in ovarian cancer cells

**Fig. 6:**
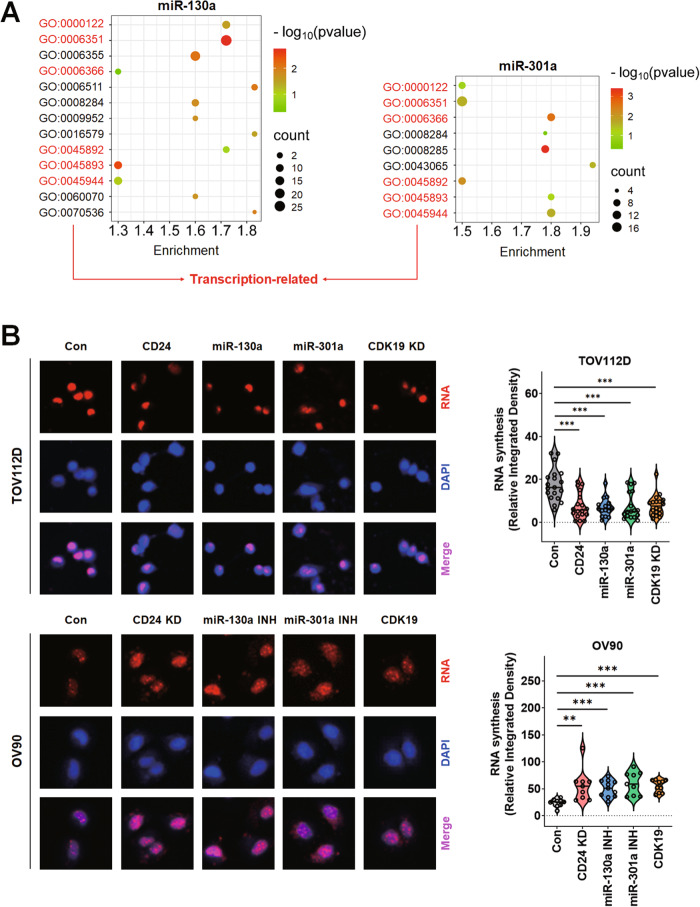
RNA synthesis regulation by CD24-miR-130a/301a-CDK19 signaling axis in ovarian cancer cells. **A** Functional annotation analysis of the putative target genes of miR-130a and 301a. **B** Analysis of CD24, miR-130a, miR-301a, or CDK19 expression-dependent RNA synthesis. Representative images (left). The graphs with all images’ integrated relative density values (right).

The putative target genes of miR-130a and 301a, other than CDK19, may be associated with CSC phenotypes. Therefore, biological processes were annotated with the putative target genes of miR-130a and 301a. In Fig. 6A, functional annotation analysis enriched transcription-related biological processes from the putative target genes of miR-130a and 301a. GO numbers and terms for the putative target genes of miR-130a were GO:0000122 (negative regulation of transcription from RNA polymerase II promoter), GO:0006351 (regulation of transcription, DNA-templated), GO:0006366 (transcription from RNA polymerase II promoter), GO:0045892 (negative regulation of transcription, DNA-templated), GO:0045893 (positive regulation of transcription, DNA-templated), and GO:00045944 (positive regulation of transcription from RNA polymerase II promoter). GO numbers and terms for miR-301a were GO:0000122 (negative regulation of transcription from RNA polymerase II promoter), GO:0006351 (transcription, DNA-templated), GO:0006366 (transcription from RNA polymerase II promoter), GO:0045892 (negative regulation of transcription, DNA-templated), GO:0045893 (positive regulation of transcription, DNA-templated), and GO:0045944 (positive regulation of transcription from RNA polymerase II promoter). The putative target genes of miR-199a-3p, 199a-5p, and 34b* were also enriched to transcription-related biological processes (Supplementary Fig. S6). Based on the functional annotation, RNA synthesis was analyzed in TOV112D and OV90 cells. In Fig. 6B, the overexpression of CD24, miR-130a, and miR-301a and the knockdown of CDK19 significantly decreased RNA synthesis in TOV112D cells compared to the control. On the other hand, the knockdown of CD24 and the overexpression of miR-130a INH, miR-301a INH, and CDK19 significantly increased RNA synthesis in OV90 cells.

### CD24-miR-130a/301a-CDK19 signaling axis could be a prognostic marker for or potential therapeutic target against ovarian cancer recurrence

**Fig. 7:**
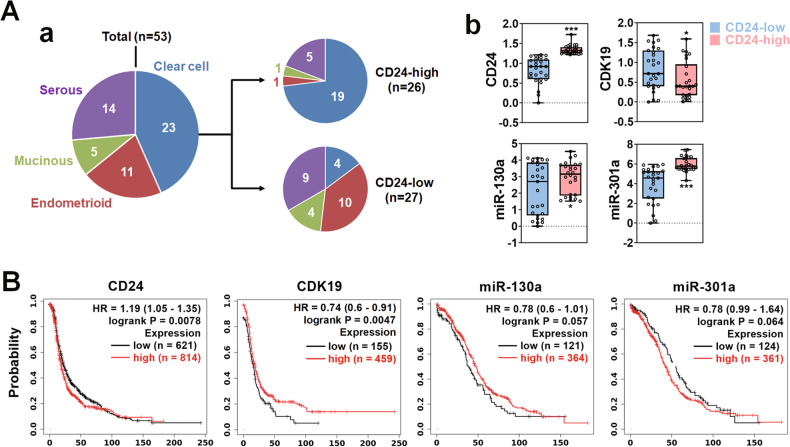
Expression of the CD24-miR-130a/301a-CDK19 signaling axis components in patient tissues and their relation with patient survival. **A** CD24-dependent expression of miR-130a, miR-301a, and CDK19 in ovarian cancer patient tissues. **a** Venn diagram presentation of sub-type distribution in the ovarian cancer patient tissue pool. The patient tissues were grouped into CD24-high and low. **b** Expression comparison analysis of miR-130a, miR-301a, and CDK19 between CD24-low and high ovarian cancer patient tissues. **B** Survival comparison analysis between ovarian cancer patient groups with low and high expression of CD24, CDK19, miR-130a, and miR-301a. Survival graphs were plotted using the KM plotter.

CD24-miR-130a/301a-CDK19 signaling axis was associated with CSC-associated phenotypes in ovarian cancer cells. Therefore, it could be a prognostic marker for or a potential therapeutic target against ovarian cancer recurrence. Therefore, the expression of CD24, miR-130a, miR-301a, and CDK19 was analyzed with 53 cases of ovarian cancer patient tissues. Patient tissues were sorted according to CD24 transcript expression level and divided in half. In Fig. 7A-a, there were 4 subtypes in the ovarian cancer tissue pool: 23 cases of clear cell, 11 cases of endometrioid, 5 cases of mucinous, and 14 cases of serous. The CD24-high group had 26 cases and included 19 cases of clear cell, 1 case of endometrioid, 1 case of mucinous, and 5 cases of serous. On the other hand, the CD24-low group had 27 cases and included 4 cases of clear cell, 10 cases of endometrioid, 4 cases of mucinous, and 9 cases of serous. In transcript expression analysis with patient tissues, CDK19 expression was lower in the CD24-high group than the CD24-low one, while miR-130a and 301a expression were higher (Fig. 7A-b). The components of the CD24-miR-130a/301a-CDK19 signaling axis may be correlated with the poor prognosis of ovarian cancer patients. Therefore, the patient survival associated with the expression of CD24, miR-130a, miR-301a, and CDK19 was analyzed. In Fig. 7B, the patient group with high CD24 expression showed a lower survival than that with low CD24 expression, while the patient group with high CDK19 expression showed a higher survival than that with low CDK19 expression. The expression levels of miR-130a and 301a were not significantly associated with patient survival.

## Discussion

**Fig. 8:**
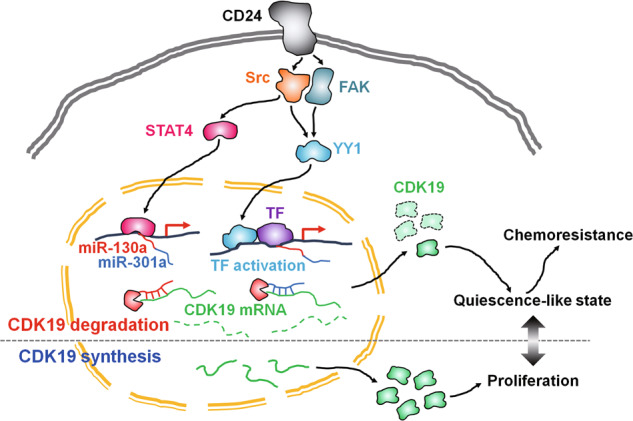
CD24-induced signaling routes for cellular quiescence-like state and chemoresistance in ovarian cancer cells. CDK19 plays a role in cell proliferation in ovarian cancer by being expressed in the absence of CD24 signaling. However, in the presence of CD24 signaling, miR-130a and 301a are expressed through Src-mediated STAT4 activation and Src and FAK-mediated YY1 activation. This results in the degradation of CDK19 mRNA, causing ovarian cancer cells to enter a quiescence-like state and become resistant to chemotherapy.

This study demonstrated that CD24 induced the expression of miR-130a and 301a via Src or FAK-mediated STAT4 and YY1 phosphorylation in ovarian cancer cells, which led to cellular quiescence-like state and chemoresistance (Fig. 8).

CD24 plays different roles in various cancers. Notably, CD24 expression does not directly correlate with the manifestation of CSC phenotypes. In breast cancer, the absence of CD24 is associated with a CSC phenotype [20]. On the other hand, despite the continued ambiguity of the relationship between CD24 expression and CSC phenotypes in ovarian cancer, CD24 expression correlated with CSC phenotypes such as tumorigenesis and drug resistance [1, 21, 22]. According to our results, CD24 expression increased G0/G1 phase cell population in ovarian cancer, which seemed to be mediated by decreased CDK19 expression and RNA synthesis. CSCs are hardly distinguished for the lack of distinct markers and phenotypes [23], but they can be characterized by low RNA content [24]. CDK19 inhibition induced G1/S transition in prostate cancer cells [25]. The decline in mRNA levels during G1 phase may be associated with RNA synthesis [26]. Unlike our results, CD24 knockdown inhibited cell cycle progression from G1 to S phase in MCF7 cells. However, CD24 ablation decreased EMT, a biological process related to CSC phenotypes [27]. This may support our finding that CD24 expression is associated with CSC phenotypes.

CD24 may regulate the manifestation of CSC phenotypes through miRNA expression. miR-130a and 301a, as members of miR-130 family miRNAs, share common seed sequences and perform similar biological functions. In our study, miR-130a and 301a targeted CDK19, and their putative target genes were associated with RNA transcription. The overexpression of miR-130a and 301a led to cellular quiescence in ovarian cancer cells and, subsequently, a cause for resistance to cisplatin and carboplatin. CSCs tend to stay in a quiescent state for survival under conditions of environmental stresses [28]. Cellular quiescence is one of the causes of treatment resistance [29]. There has been no previous report about the relationship between the expression of miR-130a/301a and cellular quiescence, but there were some reports about their correlation with chemoresistance. miR-130a was overexpressed in platinum-resistant ovarian cancer [30]. miR-130a decreased the sensitivity of ovarian cancer cells (A2780) to cisplatin [31]. miR-301a regulated glioma pathogenesis-related protein 1 (GLIPR1) expression in non-small cell lung cancer, which contributed to cisplatin resistance [32]. CSC is a significant cause of ovarian cancer with a high recurrence rate after initial treatment [33], and targeting CSC using miRNA therapeutics has been considered one of the strategies to overcome cancer recurrence [34]. Interestingly, the expression level of miR-130a and 301a was not significantly associated with patient survival (Fig. 7B). This may mean that treatment with miR-130a and 301a inhibitors effectively suppresses miR-130a/301a-dependent CDK19 downregulation (CSC phenotype manifestation) but does not affect patient survival. Therefore, miR-130a and 301a may be potential therapeutic targets for ovarian CSCs.

In annotation analysis, the putative target genes of miR-199a-3p, 199a-5p, and 34b* were also enriched to RNA transcription-related biological functions. However, they were not experimentally proven to induce CSC phenotypes. According to previous reports, miR-199a-3p increased the sensitivity of ovarian cancer cells to cisplatin [35]. miR-199a-5p suppressed the proliferation and invasion of ovarian cancer cells [36]. miR-34b* (34b-5p), a member of miR-34 family, is downregulated in cancer compared to normal and is regarded as a tumor-suppressive miRNA and therapeutic candidate in cancer [37]. Considering their roles in cancer, the CD24-induced upregulation of miR-199a-3p, 199a-5p, and 34b* was not explained in the context of CSC phenotype acquisition.

CD24 plays a dual role in the growth of ovarian cancer cells. It can either promote or inhibit cell proliferation [11, 22]. The functional properties of CD24 and the genetic or molecular heterogeneity of the cancer cells could cause this dual effect. CD24, as both a receptor and a ligand, may interact with various signaling molecules during intracellular signaling [38] and induce juxtacrine signaling in neighboring cells. Hence, the signaling outcomes of CD24 may differ depending on the specific context in which it functions. It was reported that a single receptor can have dual functions based on its molecular basis. CD244, a member of the signaling lymphocytic activation molecule family of receptors, has opposing functions depending on factors such as the degree of receptor expression, the extent of its ligation, and the relative abundance of certain adaptor molecules [39]. Ovarian cancer is a group of tumors with distinct molecular characteristics [40]. Therefore, like CD244, CD24 is also likely to exhibit dual opposing functions depending on the molecular basis of ovarian cancer cells.

In our study, the overexpression of BMI1, CD34, CD44, CTNNA1, NES, POU5F1, and THY1 was induced in TOV112D cells by CD24 overexpression and OV90 cells by CD24 knockdown (Supplementary Fig. S1). This may be explained as follows. CD24 overexpression can increase the expression of BMI1, CD34, CD44, CTNNA1, NES, POU5F1, and THY1 genes by activating direct and indirect mechanisms of gene expression. This can be achieved by producing transcription factors, activating signal pathways leading to a cascade of gene expression, and epigenetic modifications. Conversely, CD24 knockdown can increase the expression of BMI1, CD34, CD44, CTNNA1, NES, POU5F1, and THY1 genes due to the loss of feedback inhibition or compensatory upregulation. These opposing mechanisms may be activated due to different molecular contexts between TOV112D and OV90 cells. These two cells were classified into different subtypes of ovarian cancer cells and showed different mutations and gene expression [41].

## Conclusions

This study showed the relationship between CD24-regulated miRNAs and CSC phenotype acquisition in ovarian cancer and how CD24 contributes to CSC phenotype acquisition in ovarian cancer via miR-130a and 301a-dependent downregulation of CDK19. Our results suggest that the CD24-miR-130a/301a-CDK19 signaling axis could be a prognostic marker for or a potential therapeutic target against ovarian cancer recurrence.

## Materials and methods

### Patient tissue acquisition, cell culture, and stable cell establishment

Primary ovarian cancer cell clones were isolated from the mucinous cystadenocarcinoma tissue of a 46-year-old woman. The primary ovarian cancer cell clones and ovarian cancer cell lines, TOV112D and OV90 cells, were maintained in the RPMI-1640 (Invitrogen, Carlsbad, CA) supplemented with 10% fetal bovine serum (FBS) and 100 units/ml penicillin-streptomycin (Gibco BRL, Grand Island, NY). For stable cell establishment, TOV112D and OV90 cells were transduced with the lentivirus-based vectors [the pLenti-puro plasmid (Addgene, Watertown, MA) cloned with CD24 and CD24 shRNA plasmid (Origene, Rockville, MD)]. Next, the cells were treated with puromycin (Gibco BRL, Grand Island, NY) for drug selection and subjected to limiting dilution for clonal selection. The cell lines were authenticated by STR profiling, and no mycoplasma contamination was detected.

### Flow cytometry analysis

For CD24 expression level analysis, cells were stained with anti-CD24 (555427, BD Biosciences, East Rutherford, NJ). For cell cycle analysis, cells were harvested, washed with PBS, and fixed with 70% ethanol for 30 minutes. After discarding the ethanol, the cells were treated with ribonuclease (50 µl of 100 µg/ml, ThermoFisher Scientific, Waltham, MA) and added with propidium iodide (50 µg/ml, ThermoFisher Scientific, Waltham, MA). Flow cytometry analysis was performed using a FACSCalibur™ flow cytometer (BD Biosciences, East Rutherford, NJ).

### Microarray and target gene prediction

Total RNA was extracted from cells utilizing an RNeasy Protect Mini Kit (Qiagen, Hilden, Germany) according to the manufacturer’s protocol. The total RNA samples (100 ng) underwent labeling with Cyanine 3-pGp (Cy3) through the Agilent miRNA Complete Labeling and Hyb Kit (Agilent Technologies, Santa Clara, CA). Subsequently, the labeled samples were placed on an Agilent Human miRNA v15 (AMDID 029297) slide and covered with the gasket slide (Agilent Technologies, Santa Clara, CA). The hybridization process lasted for 20 hours at 55 °C using the Agilent hybridization system (Agilent Technologies, Santa Clara, CA). After hybridization, the slides were washed at room temperature (RT) in GE Wash Buffer 1 and GE Wash Buffer 2 (Agilent Technologies, Santa Clara, CA) for 5 minutes each, followed by a 20-second centrifugation at 3000 rpm to dry. The miRNA arrays were subjected to analysis using GeneSpring GX v11 (Agilent Technologies, Santa Clara, CA). The data were subjected to standard normalization methods for one-channel microarrays, which included background subtraction and percentile median normalization. Fold-change values were computed for unpaired comparisons with control samples and then averaged to obtain the mean fold-change. To identify significant changes (*P* value < 0.05), Welch’s *t* test was applied. The target gene prediction of miRNAs was performed with miRDB, miRWalk, and TargetScan.

### Real-time PCR analysis

Total RNA was extracted using Trizol (Invitrogen, Carlsbad, CA), and miRNA was isolated using miRNeasy kit (Qiagen, Hilden, Germany) according to the manufacturer’s protocols. The total RNA was subjected to reverse transcription using HyperScript^TM^ RT Master Mix (GeneAll, Seoul, Korea). For miRNA cDNA synthesis, miRNA was added with poly(A) tail using E. coli Poly(A) polymerase (E-PAP; New England Biolabs, Ipswich, MA) and then reverse transcribed, as described in a previous study [42]. For real-time PCR, 25 ng of the resulting cDNA was amplified using LaboPass^TM^ SYBR Green Q Master (Cosmogenetch, Seoul, Korea) and primers on a CFX Connect Real-Time PCR Detection System (Bio-Rad Laboratories, Hercules, CA). Primer sequences were provided in Supplementary Table S1. The expression of gene transcripts was normalized to the geometric mean of glyceraldehyde-3-phosphate dehydrogenase (GAPDH), Succinate Dehydrogenase Complex Flavoprotein Subunit A (SDHA), and Hypoxanthine Phosphoribosyltransferase 1 (HPRT1) expression. The expression level of miRNA transcripts was normalized to RNU6B expression. The relative expression levels of gene and miRNA transcripts were calculated using the ΔΔCt method.

### Gene manipulation and inhibitor treatment

CD24 and CDK19 overexpression plasmids were constructed by cloning their PCR products into pLenti-puro plasmid (Addgene, Watertown, MA). Other plasmids were purchased from companies as follows: CD24 shRNA plasmid (Origene, Rockville, MD), miR-199a-3p, miR-34c, miR-199a-5p, miR-130a, miR-301a, miR-214, miR-34b* (Applied Biological Materials, Richmond, BC, Canada), miR-199a inhibitor, miR-130a inhibitor, miR-301a inhibitor, miR-34b* inhibitor (GeneCopoeia, Rockville, MD), and CDK19 shRNA plasmid (TRCN0000003140). Transfection was performed using Lipofectamine® LTX with Plus Reagent (Invitrogen, Carlsbad, CA) according to the manufacturer’s instruction. Src phosphorylation was inhibited by treating with Src inhibitor-1 (5 µM, Sigma-Aldrich, St. Louis, MO), and FAK phosphorylation was inhibited by treating with Focal Adhesion Kinase Inhibitor-1 (5 µM, Calbiochem, San Diego, CA).

### Firefly luciferase reporter constructs and dual luciferase assays

Wild-type CDK19 3’UTR was amplified using MCF7 genomic DNA. Seed sequence-deletion mutant CDK19 3’ UTRs were amplified using an overlap extension PCR method. The wild-type and mutant 3’ UTRs were inserted downstream of the firefly luciferase-coding gene at the XbaI site in the pGL3 control vector. The authenticity and correct orientation of the inserts were verified through sequencing. For luciferase assays, transfection mixtures comprising 200 ng of firefly luciferase reporter plasmid, 10 ng of Renilla luciferase reporter plasmid (Promega, Madison, WI), and 500 ng of miRNA (miR-130a or 301a) were introduced into 293 T cells (2×10^5^ cells) in 6-well plates (SPL Life Sciences, Pocheon-si, Korea) using Lipofectamine® LTX with Plus Reagent (Invitrogen, Carlsbad, CA). The cells were harvested 48 hours after transfection. Luciferase activity was quantified from the cell lysates using a dual-luciferase reporter assay system (Promega, Madison, WI).

### Putative transcription factor binding site identification and chromatin immunoprecipitation

Putative transcription factors (TF) binding to MIR130A and MIR301A promoters were analyzed in the CpG islands of their upstream 4 kb using MethPrimer [43], PRMO [44], and BDGP [45]. TF candidates were limited to the TFs found in the BDGP-predicted regions among the PROMO-predicted TFs. The Chromatin immunoprecipitation (ChIP) assay was conducted using Pierce Agarose ChIP Kit (ThermoFisher Scientific, Waltham, MA) according to the manufacturer’s instructions. Briefly, cells were fixed using formaldehyde, washed with ice-cold PBS, and centrifuged. After removing the PBS, the cells were lysed and treated with Micrococcal Nuclease (10U/ul) for chromatin digestion for 15 minutes at 37 °C. For immunoprecipitation (IP), anti-STAT4 (ab68156, Abcam, Cambridge, UK) and anti-YY1 antibodies (ab245365, Abcam, Cambridge, UK) were added to the supernatants containing the digested chromatin and incubated overnight at 4 °C on an Adjustable-Angle Rotator (FINEPCR, Gunpo-si, Korea). Subsequently, the IP mixtures were added with ChIP Grade Protein A/G plus Agarose (Abcam, Cambridge, UK) and then incubated overnight at 4 °C with rotation. After incubation, DNAs were eluted using columns and then recovered. Putative binding site enrichment by ChIP assay was analyzed using real-time PCR with the primers provided in Supplementary Table S2.

### Western blot analysis

For sample preparation, cells were treated with PRO-PREP Protein Extraction Solution (iNtRON Biotechnology, Seongnam-si, Korea) and incubated at 4 °C for 30 minutes. Subsequently, the samples were centrifuged at 15,000 x g for 30 minutes using the Centrifuge 581 R (Eppendorf, Hamburg, Germany). After protein quantification with Protein Assay Kit (Bio-Rad Laboratories, Hercules, CA), 20 μg of protein was subjected to electrophoresis on 10% polyacrylamide gels. The separated proteins were then transferred to a PVDF membrane (Millipore Corporation, Billerica, MA) and probed with primary antibodies against CD24 (ab179821, Abcam, Cambridge, UK), Src (ab133283, Abcam, Cambridge, UK), phospho-Y419 Src (ab185617, Abcam, Cambridge, UK), phospho-Y529 Src (ab194739, Abcam, Cambridge, UK), STAT4 (ab68156, Abcam, Cambridge, UK), phospho-Y693 STAT4 (ab28815, Abcam, Cambridge, UK), YY1 (ab245365, Abcam, Cambridge, UK), CDK19 (ab168633, Abcam, Cambridge, UK), phospho-S365 YY-1 (PA5-114676, ThermoFisher Scientific, Waltham, MA), FAK (sc-271126, Santa Cruz Biotechnologies, Dallas, TX), phospho-Y397 FAK (sc-81493, Santa Cruz Biotechnologies, Dallas, TX), and GAPDH (sc-47724, Santa Cruz Biotechnologies, Dallas, TX). The primary antibodies were detected using horseradish peroxidase (HRP)-conjugated secondary antibodies (anti-mouse: SA001, anti-rabbit: SA002, GeneDepot, Katy, TX) and visualized on C-DiGit Blot Scanner (LI-COR Biosciences, Lincoln, NE) using WESTSAVE ECL Solution (AbFrontier, Seoul, Korea).

### Colony formation assay

Cells were collected using TrypLE™ Express enzyme (ThermoFisher Scientific, Waltham, MA), counted with LUNA-FX7 (Logos Biosystems, Gyeonggi-do, South Korea), and then seeded at a density of 100 cells per well in 6-well plates (SPL Life Sciences, Pocheon-si, Korea). Subsequently, the plates were incubated at 37 °C. Colonies were fixed with 100% methanol for 20 minutes, stained with crystal violet, and rinsed with water. The plates were inverted onto a tissue to air-dry overnight. The images of colonies were obtained using a BX53 system microscope (Olympus, Tokyo, Japan).

### Half-maximal inhibitory concentration determination

Cells were seeded at a density of 5 × 10^3^ cells per well in 96-well plates (SPL Life Sciences, Pocheon-si, Korea). The following day, the cells were treated with cisplatin or carboplatin half-serially diluted from 100 μM. The cells were incubated with 100 μl of serum-free RPMI containing 10 μl of Cell Counting Kit-8 (CCK-8) solution (Dojindo Molecular Technologies, Inc, Rockville, MD) for 1 hour to assess cell viability. Absorbances at 450 and 650 nm were measured using a VersaMax Microplate Reader (Molecular Devices, San Jose, CA). The Half maximal inhibitory concentration (IC50) values were calculated using Prism software based on the absorbance measurements (450–650 nm).

### RNA synthesis assay

RNA synthesis assay was performed according to the manufacturer’s instructions. Briefly, 1× RNA label dye was added to cells and incubated in a tissue culture hood for 1 hour. After discarding the medium containing RNA label dye, the cells were washed with PBS and incubated with a fixative solution for 15 minutes at RT, protected from light. The cells were washed and incubated with a permeabilization buffer for 10 minutes at RT. 1× RNA reaction cocktail was added to the cells and incubated for 30 minutes at RT. The cells were stained with DAPI and analyzed using a BX53 system microscope (Olympus, Tokyo, Japan). Integrated density was calculated using Image J version 1.53 s [46].

### Functional annotation analysis

Functional annotation analysis was performed with the putative target genes of CD24-regulated miRNAs using DAVID Functional Annotation Bioinformatic Microarray Analysis. The clustering was performed in biological processes with medium, high, and highest stringencies. The cut-off enrichment score for significant clustering was 1.3 [47]. Gene annotation with the putative target genes of CD24-regulated mRNAs was provided in Supplementary Table S3.

### Patient survival analysis

Patient survival analyses were performed using the Kaplan–Meier (KM) plotter [48]. The survival between ovarian cancer patient groups with low and high expression of CD24 or CDK19 was analyzed using the ovarian cancer mRNA dataset. The survival between ovarian cancer patient groups with low and high expression of miR-130a and 301a was analyzed using the ovarian cancer dataset of the pan-cancer.

### Graphical presentation

Cluster heatmaps, Venn diagrams, Volcano plots, and Alluvial plots were produced utilizing SRPlot (http://www.bioinformatics.com.cn/srplot). Bar graphs and Symbols with connecting line graphs were plotted using Prism software version 9.5.1 (GraphPad Software, Inc., CA). Correlation matrix heatmaps were generated using Excel’s conditional formatting feature (Microsoft, Redmond, WA).

### Statistics

The statistical significance of gene expression in cells and tissues and correlation in microarray and NGS datasets was assessed through Student’s t-test (two-tailed) and Pearson’s correlation coefficient, respectively. Results were deemed statistically significant when p < 0.05. All statistical analyses were conducted using Prism software version 9.5.1 (GraphPad Software, Inc., CA). Asterisks were employed to represent *p* values: one for *p* ≤ 0.05, two for *p* ≤ 0.01, and three for *p* ≤ 0.001.

### Supplementary information

Supplementary Information

Uncropped images of Western blot data

Reproducibility checklist

## Data Availability

The raw and processed data of the microarray are available in the GEO database (GSE164748). All data generated during the study leading to the presented findings are included in this published article and its Supplementary Data files.
