# Supplementary material for: CD24 induced cellular quiescence-like state and chemoresistance in ovarian cancer cells via miR-130a/301a-dependent CDK19 downregulation
Source: Cell Death Discov. 2024 Feb 15;10:81. doi: 10.1038/s41420-024-01858-y (PMC10869724; doi:10.1038/s41420-024-01858-y)
Supplement: Supplementary file 1 — Supplementary Information [file 41420_2024_1858_MOESM1_ESM.pdf]

Supplementary Information

Supplementary Figures and Tables

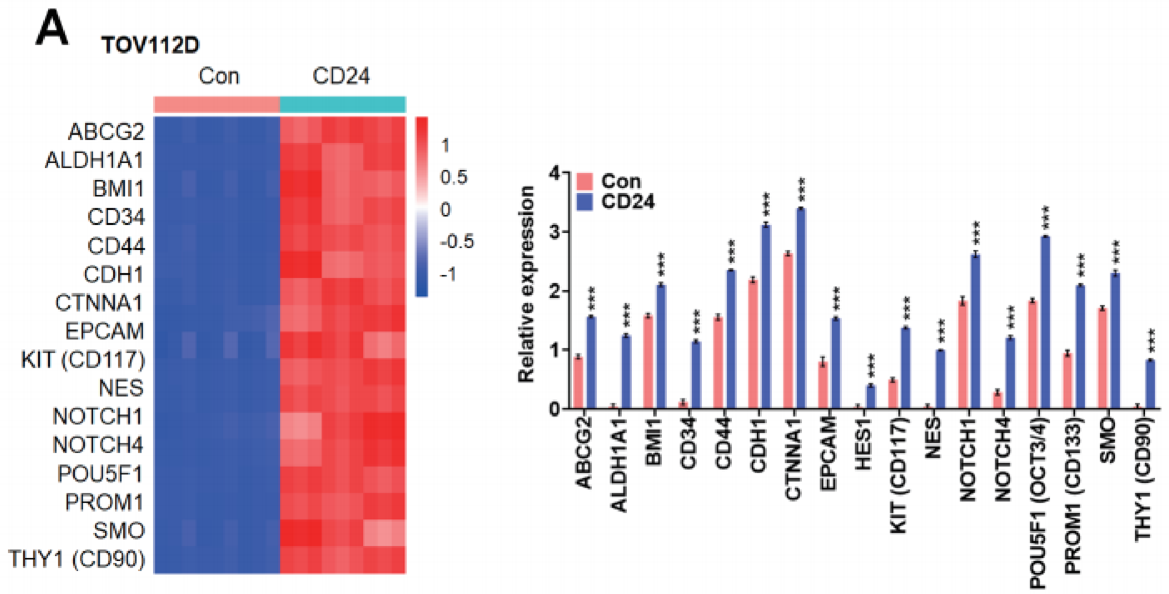

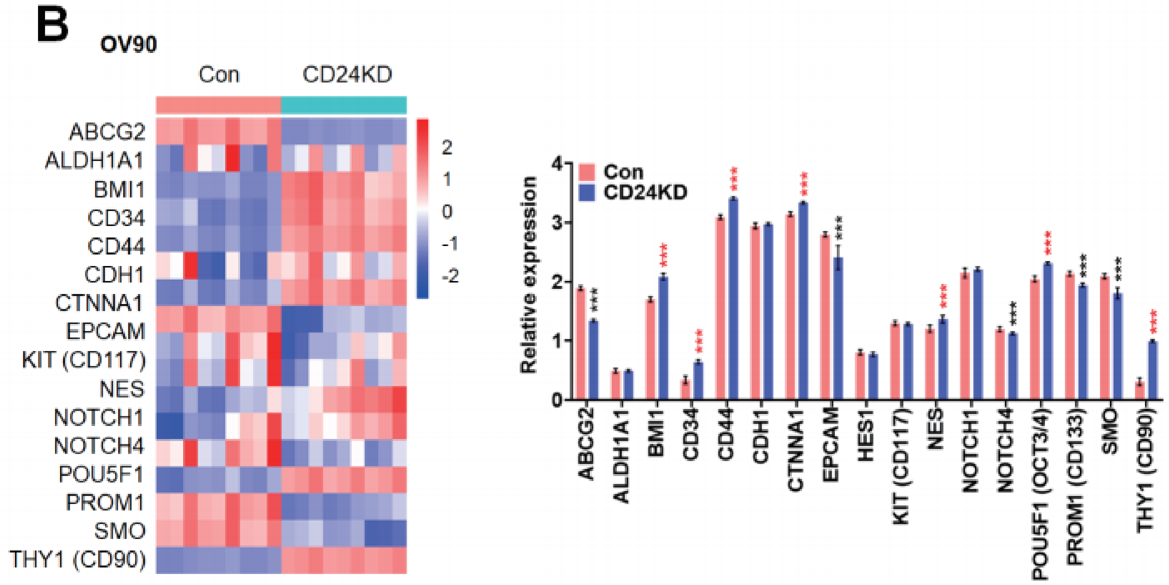

Supplementary Figure S1. Comparative analysis of stemness-related gene expression between CD24-low and high ovarian cancer cells.

Heatmap and Grouped bar graph presentation of stemness-related gene expression of A) control and CD24-overexpressing TOV112 cells, and B) control and CD24-knockdown OV90 cells.

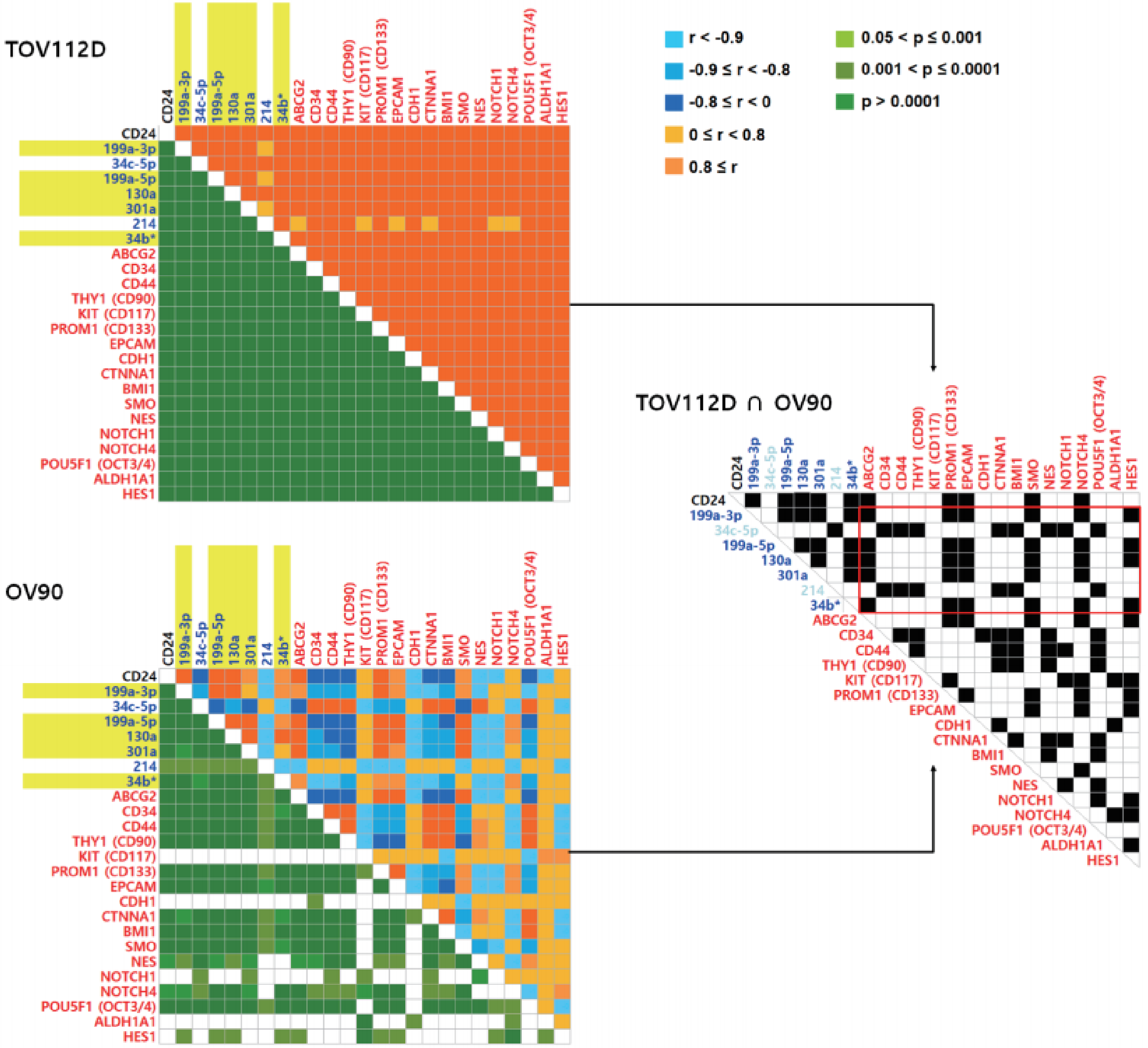

Supplementary Figure S2. Correlation between CD24, CD24-regulated miRNAs, and stemness-related genes.

Correlation matrices were plotted with correlation coefficients and p-values. Correlation coefficients were presented in upper half matrices. P-values were displayed in lower half matrices. The correlation commonly and positively shown in TOV112 and OV90 cells was presented in a separate half matrix.

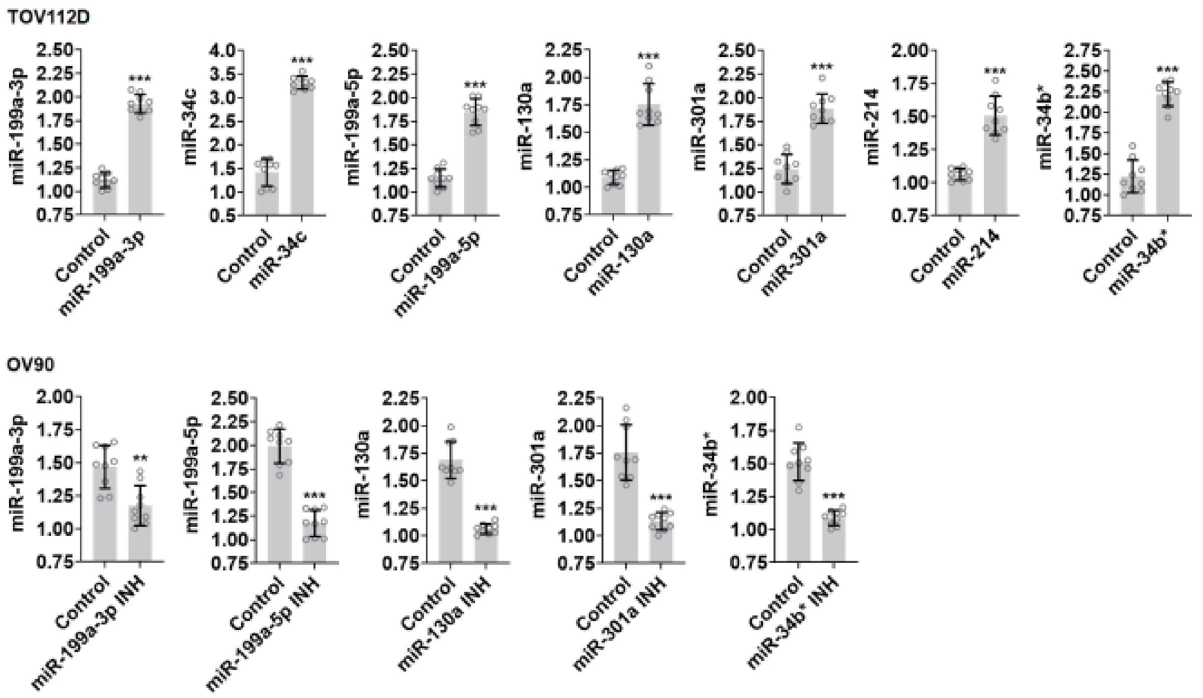

Supplementary Figure S3. The transfection efficacy evaluation of overexpression and inhibitor plasmid vectors using semi-quantitative PCR analysis.

PCR analysis was performed in triplicate and normalized with RNU6B expression.

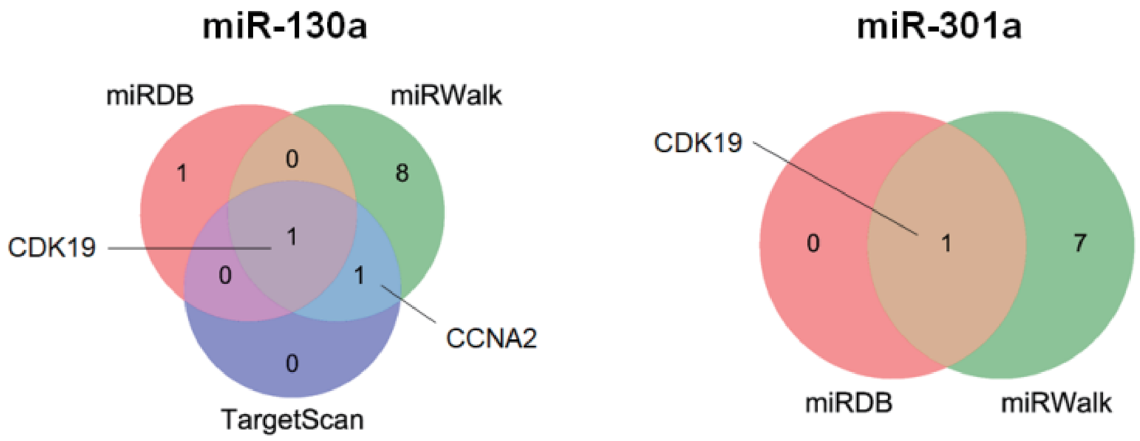

From TargetScan

miRNA	Position in the CDK19 3'UTR	Seed sequence	Seed match
miR-130a-3p	857-863	UGCACUA	7mer-1A
	1219-1225	UUGCACU	7mer-m8
	1645-1652	UUGCACUA	8mer
	3523-3529	UGCACUA	7mer-1A
miR-301a-3p	857-863	UGCACUA	7mer-1A
	1219-1225	UUGCACU	7mer-m8
	1645-1652	UUGCACUA	8mer
	3523-3529	UGCACUA	7mer-1A

Supplementary Figure S4. Putative target genes of miR-130a and 301a among cell cycle-associated genes.

The putative target genes of miR-130a and 301a were analyzed in miRDB, miRWalk, and TargetScan. CDK19 was predicted as a target gene of miR-130a commonly in miRDB, miRWalk, and TargetScan, whereas CDK19 was predicted as a target gene of miR-301a commonly in miRDB and miRWalk. Venn diagram was plotted using SRplot. The information on the putative binding position of miR-130a/301a on the CDK19 3'UTR and their seed sequences was from TargetScan.

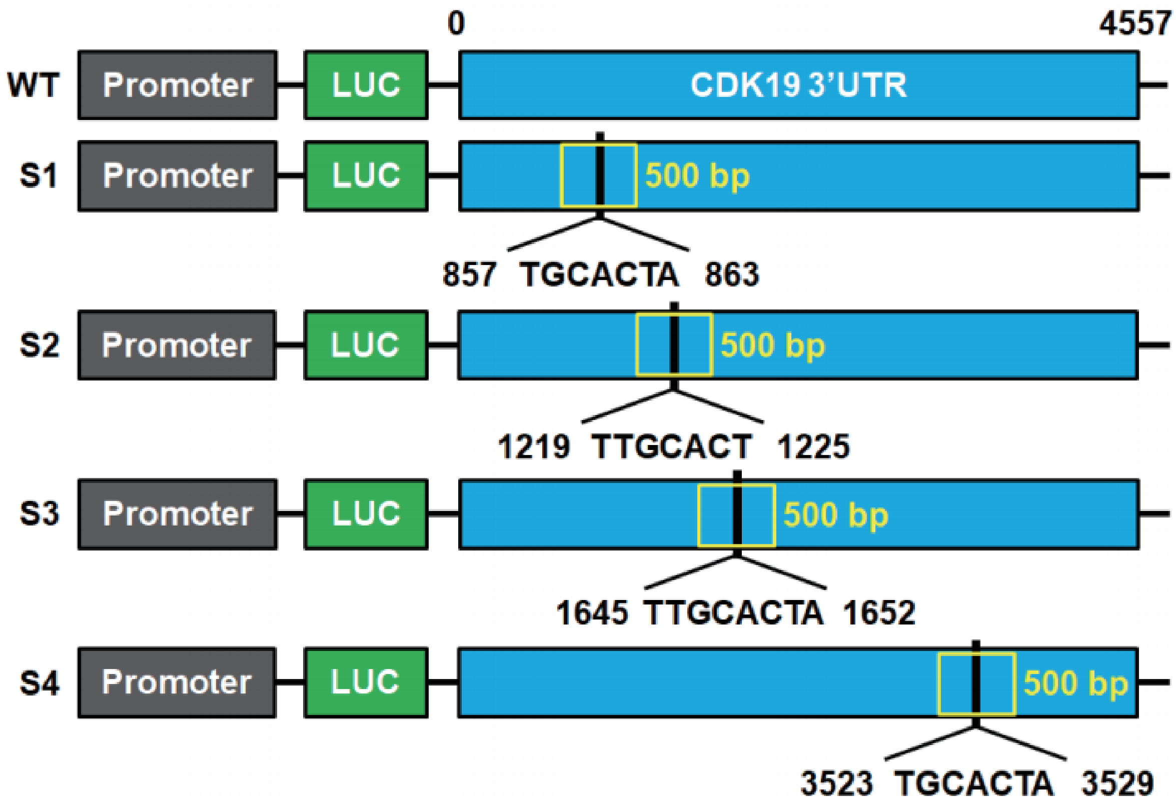

Supplementary Figure S5. Plasmid constructs of wild and deletion mutant CDK19 3'UTR.

Wild and deletion mutant CDK19 3'UTRs were cloned behind the luciferase (LUC) gene in the pGL3 control vector. The deleted sequences were the seed sequences recognized by miR-130a and 301a.

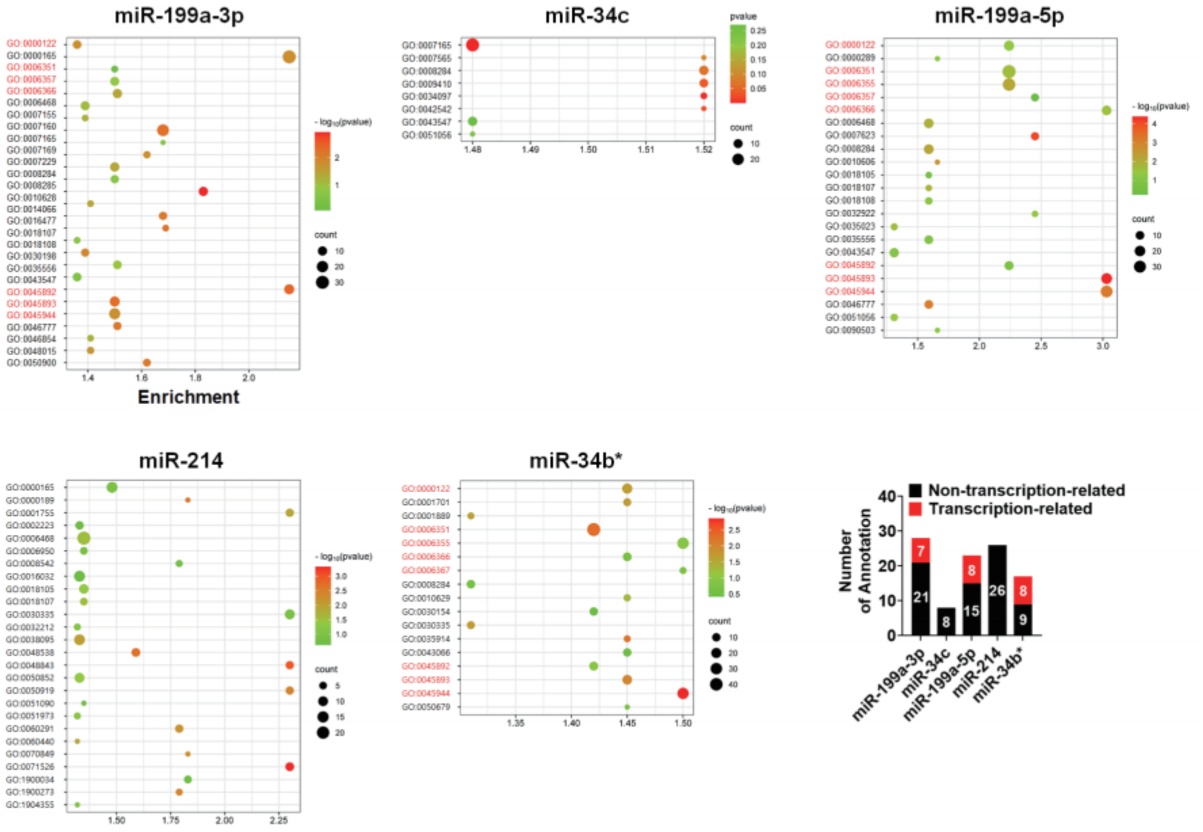

Supplementary Figure S6. Functional annotation analysis of the putative target genes of CD24-regulated miRNAs other than miR-130a and 301a.

Functional annotation analyses predicted transcription-related biological processes in the putative target genes of miR-199a-3p, 199a-5p, and miR-34b*. The predicted transcription-related biological processes were highlighted in red, with 7 for miR-199a-3p, 8 for miR-199a-5p, and 8 for miR-34b*.

Supplementary Table S1. Primers for gene expression analysis.

	Gene	Strand	Sequence (5' to 3')
**Gene expression**	CD24	Forward	GCTCCTACCCACGCAGATTT
		Reverse	CCACGAAGAGACTGGCTGTT
	CDK19	Forward	AGAACAGCACCCAGACCAAC
		Reverse	TGCTCTGGGACTGAGAGGAT
	GAPDH	Forward	TGCACCACCAACTGCTTAGC
		Reverse	GGCATGGACTGTGGTCATGAG
	HPRT1	Forward	TGACACTGGCAAAACAATGCA
		Reverse	GGTCCTTTTCACCAGCAAGCT
	SDHA	Forward	TGGGAACAAGAGGGCATCTG
		Reverse	CCACCACTGCATCAAATTCATG
	ABCG2	Forward	CACCTTATTGGCCTCAGGAA
		Reverse	CCTGCTTGGAAGGCTCTATG
	ALDH1A1	Forward	TGTTAGCTGATGCCGACTTG
		Reverse	TTCTTAGCCCGCTCAACACT
	BMI1	Forward	CCAGGGCTTTTCAAAAATGA
		Reverse	CCGATCCAATCTGTTCTGGT
	CD34	Forward	GCAAGCCACCAGAGCTATTC
		Reverse	TCCACCGTTTTCCGTGTAAT
	CD44	Forward	TCTGTGCAGCAAACAACACA
		Reverse	TAGGGTTGCTGGGGTAGATG
	CDH1	Forward	TGCCCAGAAAATGAAAAAGG
		Reverse	GTGTATGTGGCAATGCGTTC
	CTNNA1	Forward	CATCAGTGCTGCCAAGAAAA
		Reverse	TGTTCAGCTGGTGGCAGTAG
	EPCAM	Forward	GCTGGTGTGTGAACACTGCT
		Reverse	ACGCGTTGTGATCTCCTTCT
	HES1	Forward	CGGACATTCTGGAAATGACA

		Reverse	CATTGATCTGGGTCATGCAG
	KIT	Forward	TCATGGTCGGATCACAAAGA
		Reverse	AGGGGCTGCTTCCTAAAGAG
	NES	Forward	AACAGCGACGGAGGTCTCTA
		Reverse	TTCTCTTGTCCCGCAGACTT
	NOTCH1	Forward	ACTGTGAGGACCTGGTGGAC
		Reverse	TTGTAGGTGTTGGGGAGGTC
	NOTCH4	Forward	CTAGGGGCTCTTCTCGTCCT
		Reverse	CAACTTCTGCCTTTGGCTTC
	POU5F1	Forward	CGAAAGAGAAAGCGAACCAG
		Reverse	GCCGGTTACAGAACCACACT
	PROM1	Forward	TTGTGGCAAATCACCAGGTA
		Reverse	TCAGATCTGTGAACGCCTTG
	SMO	Forward	GGGAGGCTACTTCCTCATCC
		Reverse	GGCAGCTGAAGGTAATGAGC
	THY1	Forward	CTAGTGGACCAGAGCCTTCG
		Reverse	TGGAGTGCACACGTGTAGGT
**miRNA expression**	miR-199a-3p	Forward	ACAGTAGTCTGCACATTGGTTA
	miR-34c	Forward	AGGCAGTGTAGTTAGCTGATTGC
	miR-199a-5p	Forward	CCCAGTGTTCAGACTACCTGTTC
	miR-130a	Forward	CAGTGCAATGTTAAAAGGGCAT
	miR-301a	Forward	CAGTGCAATAGTATTGTCAAAGC
	miR-214	Forward	ACAGCAGGCACAGACAGGCAGT
	miR-34b*	Forward	TAGGCAGTGTCATTAGCTGATTG
	RNU6B	Forward	CGCTTCGGCAGCACATATAC
	URT	Reverse	AACGAGACGACGACAGACTTTTTTTTTTTT
	Universal PCR	Reverse	AACGAGACGACGACAGACTTT

Supplementary Table S2. Primers for ChIP enrichment analysis.

Name	Coverage	Strand	Sequence (5’ to 3’)	TF	
miR-130a_S1	S1	Forward	GGAACTGGATCTGCCTTCAC	STAT4	
		Reverse	CTGGCTTCTCACCTCAACAAC		
miR-130a_S2	S2	Forward	CTGACCTCGCGGACTGTT		YY1
		Reverse	AACCCTTTTGAAACGAGATCCT		
miR-130a_S3	S3	Forward	TGCTCACTCCATTCAAGAATCC	STAT4	
		Reverse	CCTCTCCTCTGCCGATCTC		
miR-130a_S4	S4	Forward	CAGGCAGAGGATCCTAACGA	STAT4	
		Reverse	CTCCCTTCCCCAAGAGAGAA		
miR-301a_S1	S1 to S5	Forward	GGAACTGATGGTGAGTGCAA	STAT4	YY1
		Reverse	GAGGGAACTCGACTCTGGTC		
miR-301a_S2	S6 to S11	Forward	CCTCGGGTAAGGGGTTACAT	STAT4	YY1
		Reverse	GTAAAGGTGAGGGCAAGGAA		
miR-301a_S3	S12, S13	Forward	CCCTCACCTTTACACCCCTTA	STAT4	YY1
		Reverse	TGTGAACCACCTCTCCCTTC		
miR-301a_S4	S14	Forward	GAAGGGAGAGGTGGTTCACA	STAT4	
		Reverse	TTTAGAACGTGAGCGGGTTG		
miR-301a_S5	S15 to S17	Forward	GGCCGAGAAAGTCTGAGAAA	STAT4	
		Reverse	CTCACACCTCTTCCCTCACC		
miR-301a_S6	S18, S19	Forward	TTGAGACAGGAAAGGCATCC	STAT4	
		Reverse	CAAACACGCCAGCAACAA		
miR-301a_S7	S20 to S22	Forward	TGTCTTCAGCCCTTTTCGTT	STAT4	
		Reverse	GCTTCCTCCAAAGTCCAGGT		
miR-301a_S8	S23, S24	Forward	TTGAGACTACCCTGGCCAAC		YY1
		Reverse	CTCCCGAGTAGCTGGGACTA		

Supplementary Table S3. Gene annotation of the putative target genes of CD24-regulated miRNAs.

Transcription-related biological processes were highlighted in red.

miRNA	Classification Stringency	Cluster	GO number	GO Term	Enrichment Score	Count	P_Value	Benjamini
199a-3p	High	1	GO:0048015	phosphatidylinositol-mediated signaling	1.41	5	0.021	0.85
			GO:0014066	regulation of phosphatidylinositol 3-kinase signaling	1.41	4	0.042	1
			GO:0046854	phosphatidylinositol phosphorylation	1.41	4	0.066	1
	Medium	1	GO:0046777	protein autophosphorylation	1.51	7	0.0073	0.55
			GO:0006468	protein phosphorylation	1.51	10	0.038	1
			GO:0035556	intracellular signal transduction	1.51	8	0.11	1
		2	GO:0030198	extracellular matrix organization	1.39	7	0.013	0.72
			GO:0007160	cell-matrix adhesion	1.39	4	0.06	1
			GO:0007155	cell adhesion	1.39	9	0.086	1
	Low	1	GO:0045892	negative regulation of transcription, DNA-templated	2.15	13	0.0041	0.55
			GO:0000122	negative regulation of transcription from RNA polymerase II promoter	2.15	16	0.0052	0.55
			GO:0006351	transcription, DNA-templated	2.15	30	0.016	0.72
		2	GO:0018107	peptidyl-threonine phosphorylation	1.69	4	0.0062	0.55
		3	GO:0050900	leukocyte migration	1.62	6	0.0073	0.55
			GO:0007229	integrin-mediated signaling pathway	1.62	5	0.017	0.72
	Lowest	1	GO:0010628	positive regulation of gene expression	1.83	10	0.0012	0.34
		2	GO:0007165	signal transduction	1.68	22	0.0053	0.55
			GO:0016477	cell migration	1.68	7	0.0073	0.55
			GO:0007169	transmembrane receptor protein tyrosine kinase signaling pathway	1.68	3	0.25	1
		3	GO:0045893	positive regulation of transcription, DNA-templated	1.5	13	0.0052	0.55
			GO:0045944	positive regulation of transcription from RNA polymerase II promoter	1.5	18	0.017	0.72
			GO:0008284	positive regulation of cell proliferation	1.5	10	0.042	1
			GO:0006366	transcription from RNA polymerase II promoter	1.5	9	0.14	1
			GO:0008285	negative regulation of cell proliferation	1.5	7	0.2	1
			GO:0006357	regulation of transcription from RNA polymerase II promoter	1.5	4	0.82	1

		4	GO:0000165	MAPK cascade	1.36	8	0.016	0.72
			GO:0018108	peptidyl-tyrosine phosphorylation	1.36	4	0.19	1
			GO:0043547	positive regulation of GTPase activity	1.36	8	0.33	1
34c-5p	Low	1	GO:0034097	response to cytokine	1.52	5	0.004	1
			GO:0042542	response to hydrogen peroxide	1.52	4	0.026	1
			GO:0009410	response to drug	1.52	9	0.037	1
			GO:0008284	positive regulation of cell proliferation	1.52	11	0.067	1
			GO:0007565	female pregnancy	1.52	4	0.1	1
	Lowest	2	GO:0007165	signal transduction	1.48	29	0.00058	0.7
			GO:0051056	regulation of small GTPase mediated signal transduction	1.48	4	0.23	1
			GO:0043547	positive regulation of GTPase activity	1.48	10	0.27	1
199a-5p	Highest	1	GO:0010606	positive regulation of cytoplasmic mRNA processing body assembly	1.66	3	0.0028	0.93
			GO:0090503	RNA phosphodiester bond hydrolysis, exonucleolytic	1.66	3	0.059	1
			GO:0000289	nuclear-transcribed mRNA poly(A) tail shortening	1.66	3	0.062	1
		2	GO:0035023	regulation of Rho protein signal transduction	1.31	5	0.027	1
			GO:0051056	regulation of small GTPase mediated signal transduction	1.31	6	0.041	1
			GO:0043547	positive regulation of GTPase activity	1.31	13	0.1	1
	Low	1	GO:0045893	positive regulation of transcription, DNA-templated	3.03	21	4.20E-05	0.069
			GO:0045944	positive regulation of transcription from RNA polymerase II promoter	3.03	28	0.00065	0.31
			GO:0006366	transcription from RNA polymerase II promoter	3.03	14	0.03	1
	Lowest	1	GO:0007623	circadian rhythm	2.45	8	8.70E-05	0.072
			GO:0032922	circadian regulation of gene expression	2.45	4	0.046	1
			GO:0006357	regulation of transcription from RNA polymerase II promoter	2.45	7	0.59	1
		2	GO:0006355	regulation of transcription, DNA-templated	2.24	34	0.0068	1
			GO:0006351	transcription, DNA-templated	2.24	39	0.024	1
			GO:0000122	negative regulation of transcription from RNA polymerase II promoter	2.24	17	0.048	1

			GO:0045892	negative regulation of transcription, DNA-templated	2.24	11	0.17	1
		3	GO:0046777	protein autophosphorylation	1.59	10	0.00074	0.31
			GO:0008284	positive regulation of cell proliferation	1.59	15	0.0064	1
			GO:0006468	protein phosphorylation	1.59	14	0.013	1
			GO:0018107	peptidyl-threonine phosphorylation	1.59	4	0.016	1
			GO:0018108	peptidyl-tyrosine phosphorylation	1.59	6	0.065	1
			GO:0035556	intracellular signal transduction	1.59	10	0.12	1
			GO:0018105	peptidyl-serine phosphorylation	1.59	4	0.26	1
130a	Medium	1	GO:0006511	ubiquitin-dependent protein catabolic process	1.83	6	0.0089	1
			GO:0070536	protein K63-linked deubiquitination	1.83	3	0.011	1
			GO:0016579	protein deubiquitination	1.83	4	0.032	1
	Low	1	GO:0045893	positive regulation of transcription, DNA-templated	1.3	11	0.0032	0.89
			GO:0045944	positive regulation of transcription from RNA polymerase II promoter	1.3	12	0.08	1
			GO:0006366	transcription from RNA polymerase II promoter	1.3	5	0.48	1
	Lowest	1	GO:0006351	transcription, DNA-templated	1.72	26	0.0018	0.76
			GO:0000122	negative regulation of transcription from RNA polymerase II promoter	1.72	11	0.029	1
			GO:0045892	negative regulation of transcription, DNA-templated	1.72	7	0.14	1
		2	GO:0006355	regulation of transcription, DNA-templated	1.6	20	0.0078	1
			GO:0008284	positive regulation of cell proliferation	1.6	9	0.016	1
			GO:0009952	anterior/posterior pattern specification	1.6	4	0.018	1
			GO:0060070	canonical Wnt signaling pathway	1.6	4	0.02	1
301a	Low	1	GO:0006366	transcription from RNA polymerase II promoter	1.8	10	0.0025	0.5
			GO:0045944	positive regulation of transcription from RNA polymerase II promoter	1.8	12	0.023	1
			GO:0045893	positive regulation of transcription, DNA-templated	1.8	7	0.072	1
	Lowest	1	GO:0043065	positive regulation of apoptotic process	1.94	6	0.028	1
		2	GO:0008285	negative regulation of cell proliferation	1.78	10	0.0004	0.29
			GO:0008284	positive regulation of cell proliferation	1.78	4	0.49	1

		3	GO:0045892	negative regulation of transcription, DNA-templated	1.5	9	0.0073	1
			GO:0006351	transcription, DNA-templated	1.5	19	0.025	1
			GO:0000122	negative regulation of transcription from RNA polymerase II promoter	1.5	8	0.11	1
214	High	1	GO:0060440	trachea formation	1.32	3	0.013	1
			GO:0051973	positive regulation of telomerase activity	1.32	4	0.061	1
			GO:0032212	positive regulation of telomere maintenance via telomerase	1.32	4	0.077	1
			GO:1904355	positive regulation of telomere capping	1.32	3	0.087	1
	Medium	1	GO:0071526	semaphorin-plexin signaling pathway	2.3	7	0.00048	0.51
			GO:0048843	negative regulation of axon extension involved in axon guidance	2.3	6	0.0011	0.55
			GO:0050919	negative chemotaxis	2.3	6	0.0038	1
			GO:0001755	neural crest cell migration	2.3	6	0.013	1
			GO:0030335	positive regulation of cell migration	2.3	10	0.12	1
		2	GO:0000189	MAPK import into nucleus	1.83	3	0.0029	0.88
			GO:0070849	response to epidermal growth factor	1.83	3	0.0056	1
			GO:1900034	regulation of cellular response to heat	1.83	5	0.21	1
		3	GO:1900273	positive regulation of long-term synaptic potentiation	1.79	4	0.0041	1
			GO:0060291	long-term synaptic potentiation	1.79	6	0.0062	1
			GO:0008542	visual learning	1.79	4	0.16	1
		4	GO:0048538	thymus development	1.59	7	0.0021	0.72
		5	GO:0038095	Fc-epsilon receptor signaling pathway	1.33	13	0.01	1
			GO:0050852	T cell receptor signaling pathway	1.33	10	0.043	1
			GO:0002223	stimulatory C-type lectin receptor signaling pathway	1.33	6	0.23	1
			GO:0016032	viral process	1.33	13	0.23	1
	Low	1	GO:0000165	MAPK cascade	1.48	13	0.12	1
	Lowest	1	GO:0006468	protein phosphorylation	1.35	23	0.03	1
			GO:0018107	peptidyl-threonine phosphorylation	1.35	5	0.03	1
			GO:0018105	peptidyl-serine phosphorylation	1.35	9	0.042	1
			GO:0006950	response to stress	1.35	5	0.12	1
			GO:0051090	regulation of sequence-specific DNA binding transcription factor activity	1.35	3	0.18	1

34*	Low	1	GO:0030335	positive regulation of cell migration	1.31	8	0.02	1
			GO:0001889	liver development	1.31	5	0.024	1
			GO:0008284	positive regulation of cell proliferation	1.31	10	0.25	1
	Lowest	1	GO:0045944	positive regulation of transcription from RNA polymerase II promoter	1.5	28	0.0014	0.73
			GO:0006355	regulation of transcription, DNA-templated	1.5	29	0.12	1
			GO:0006367	transcription initiation from RNA polymerase II promoter	1.5	5	0.19	1
		2	GO:0035914	skeletal muscle cell differentiation	1.45	5	0.0058	1
			GO:0045893	positive regulation of transcription, DNA-templated	1.45	16	0.0098	1
			GO:0000122	negative regulation of transcription from RNA polymerase II promoter	1.45	19	0.021	1
			GO:0001701	in utero embryonic development	1.45	8	0.021	1
			GO:0010629	negative regulation of gene expression	1.45	6	0.053	1
			GO:0050679	positive regulation of epithelial cell proliferation	1.45	3	0.22	1
			GO:0006366	transcription from RNA polymerase II promoter	1.45	11	0.23	1
			GO:0043066	negative regulation of apoptotic process	1.45	9	0.36	1
		3	GO:0006351	transcription, DNA-templated	1.42	44	0.0048	1
			GO:0045892	negative regulation of transcription, DNA-templated	1.42	12	0.12	1
			GO:0030154	cell differentiation	1.42	9	0.38	1
