# Supplementary figures and images for: CD24 induced cellular quiescence-like state and chemoresistance in ovarian cancer cells via miR-130a/301a-dependent CDK19 downregulation
Source: Cell Death Discov. 2024 Feb 15;10:81. doi: 10.1038/s41420-024-01858-y (PMC10869724; doi:10.1038/s41420-024-01858-y)

Figure 2B

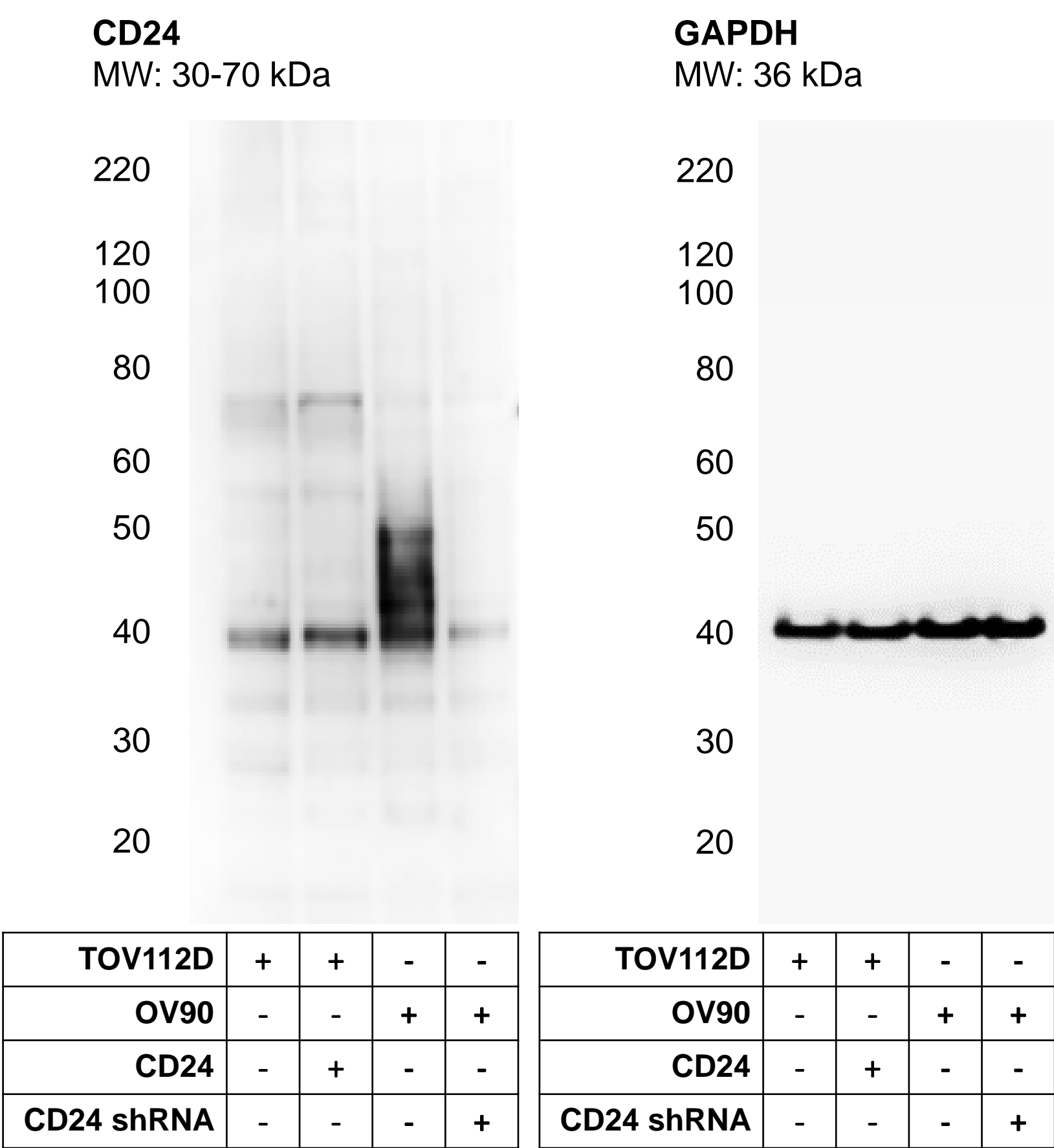

Figure 5E

TOV112D\_CD24

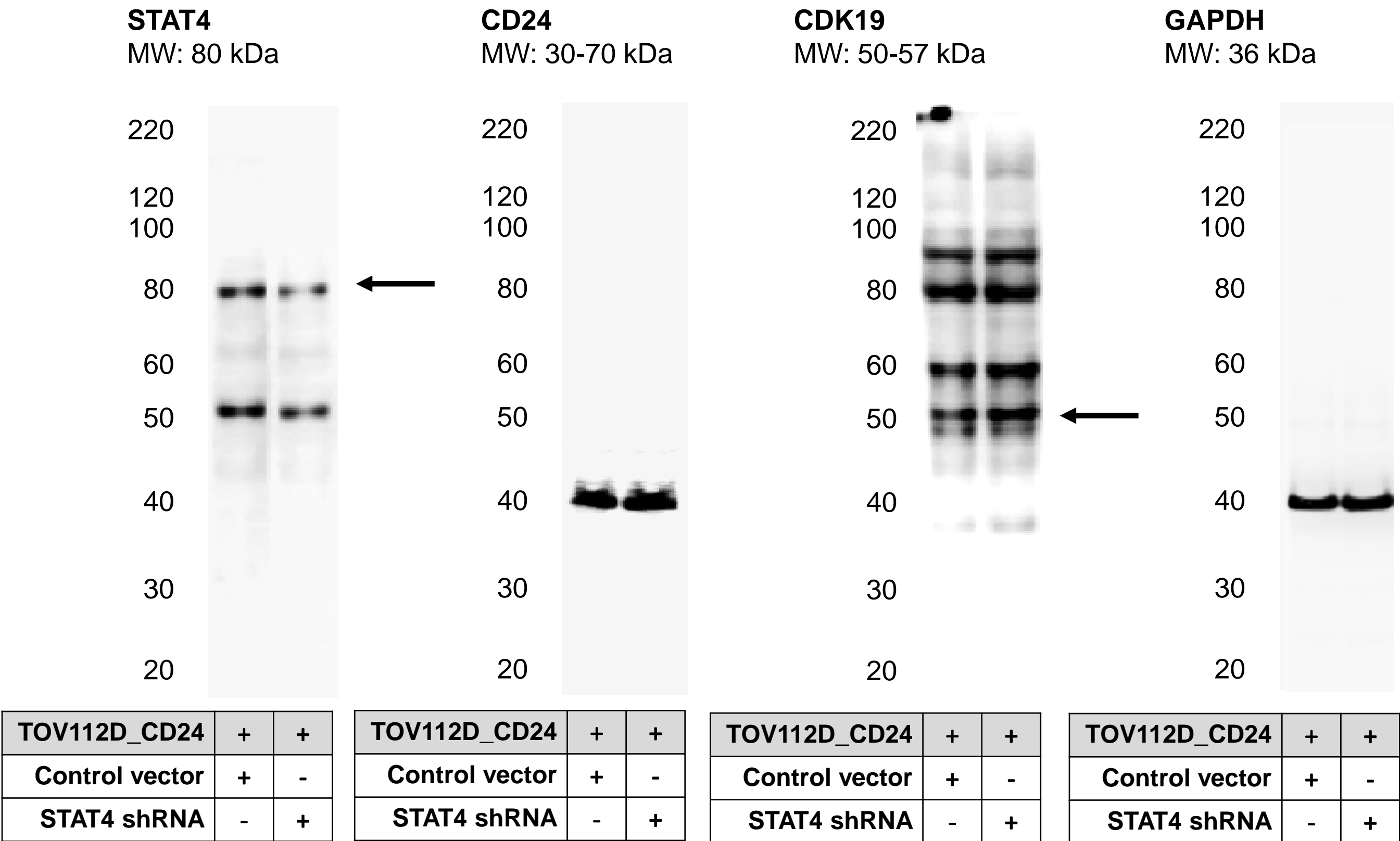

Figure 5F

TOV112D\_CD24

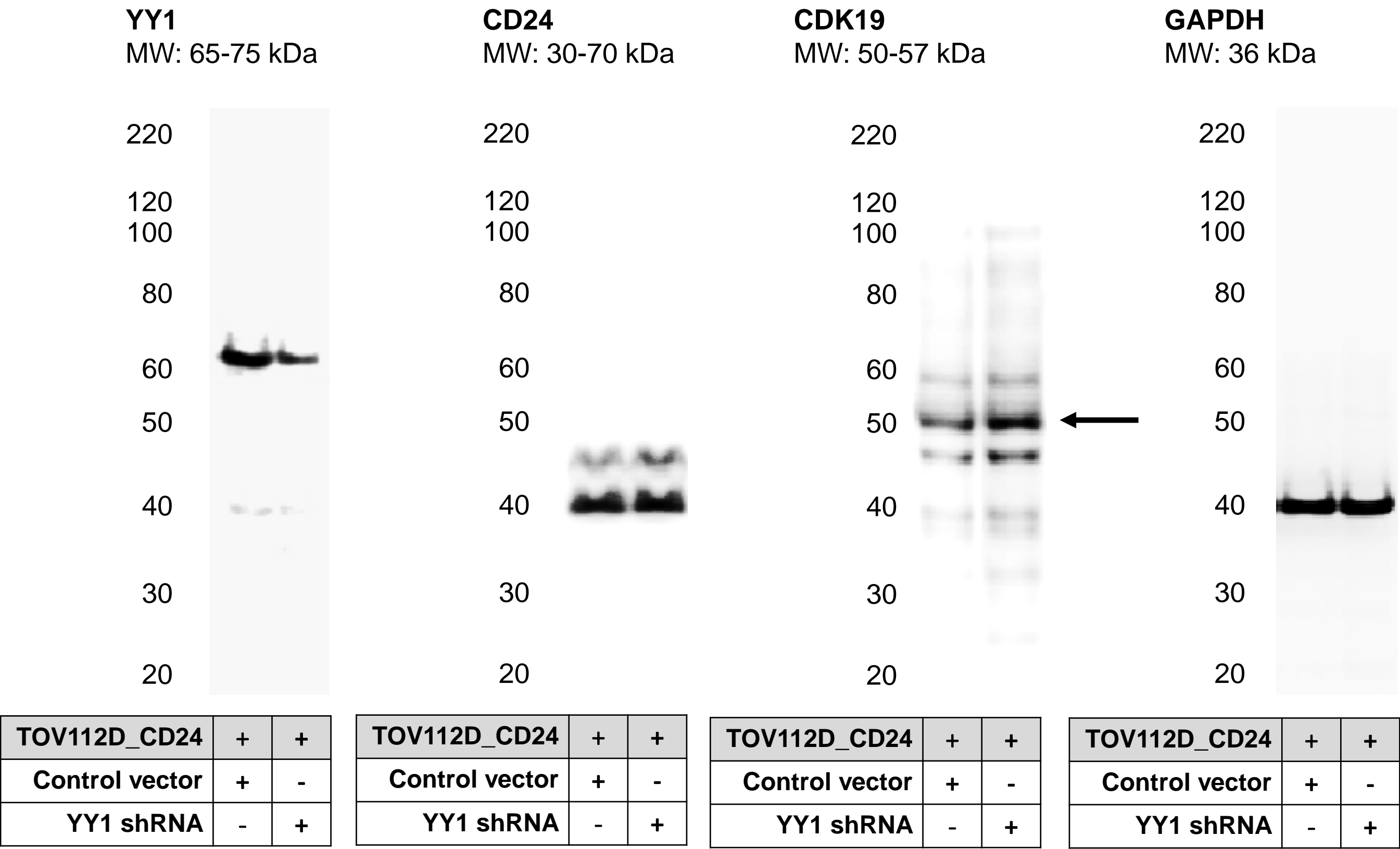

OV90

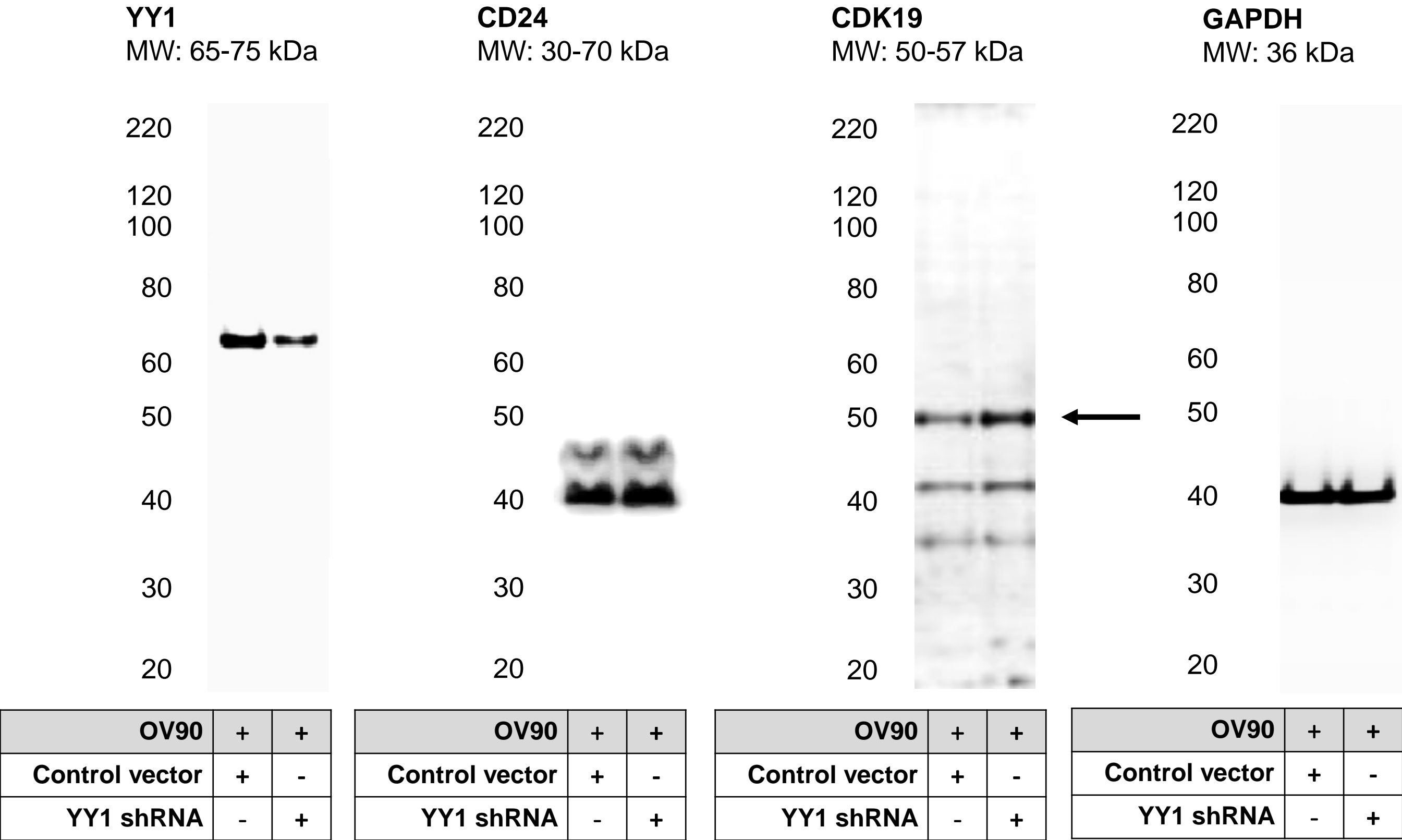

Figure 5G

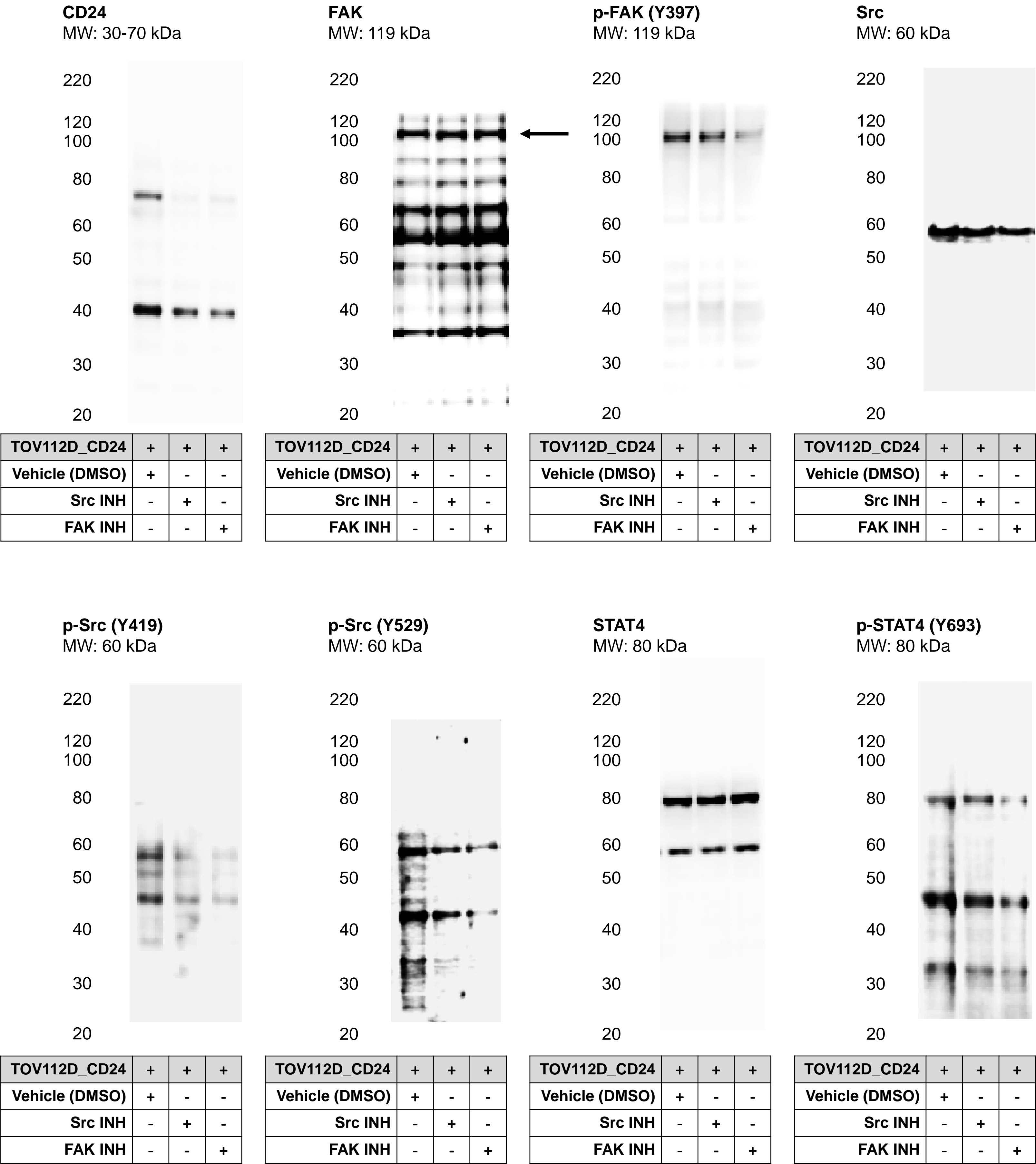

Figure 5G

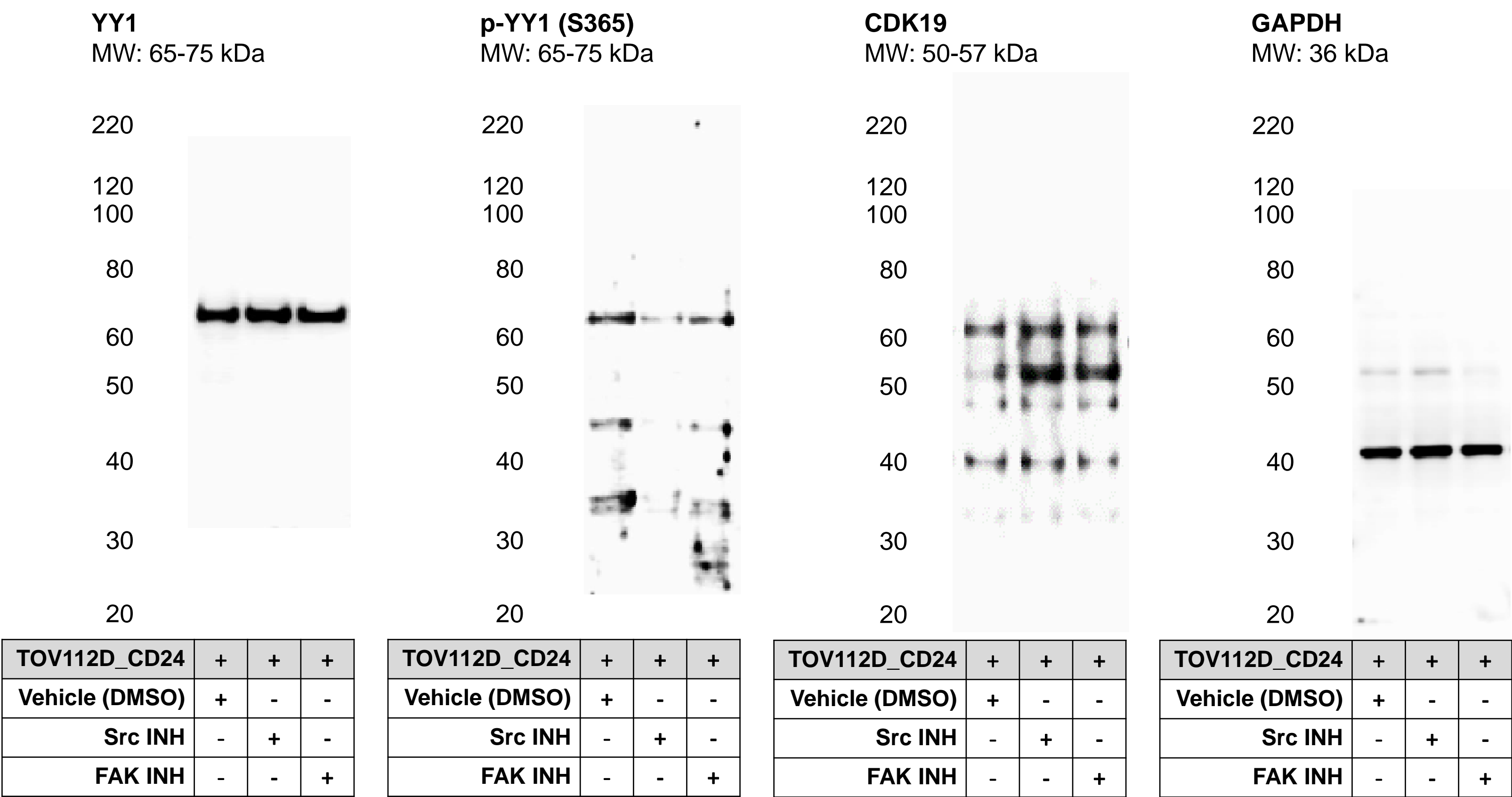

### Figure 5G

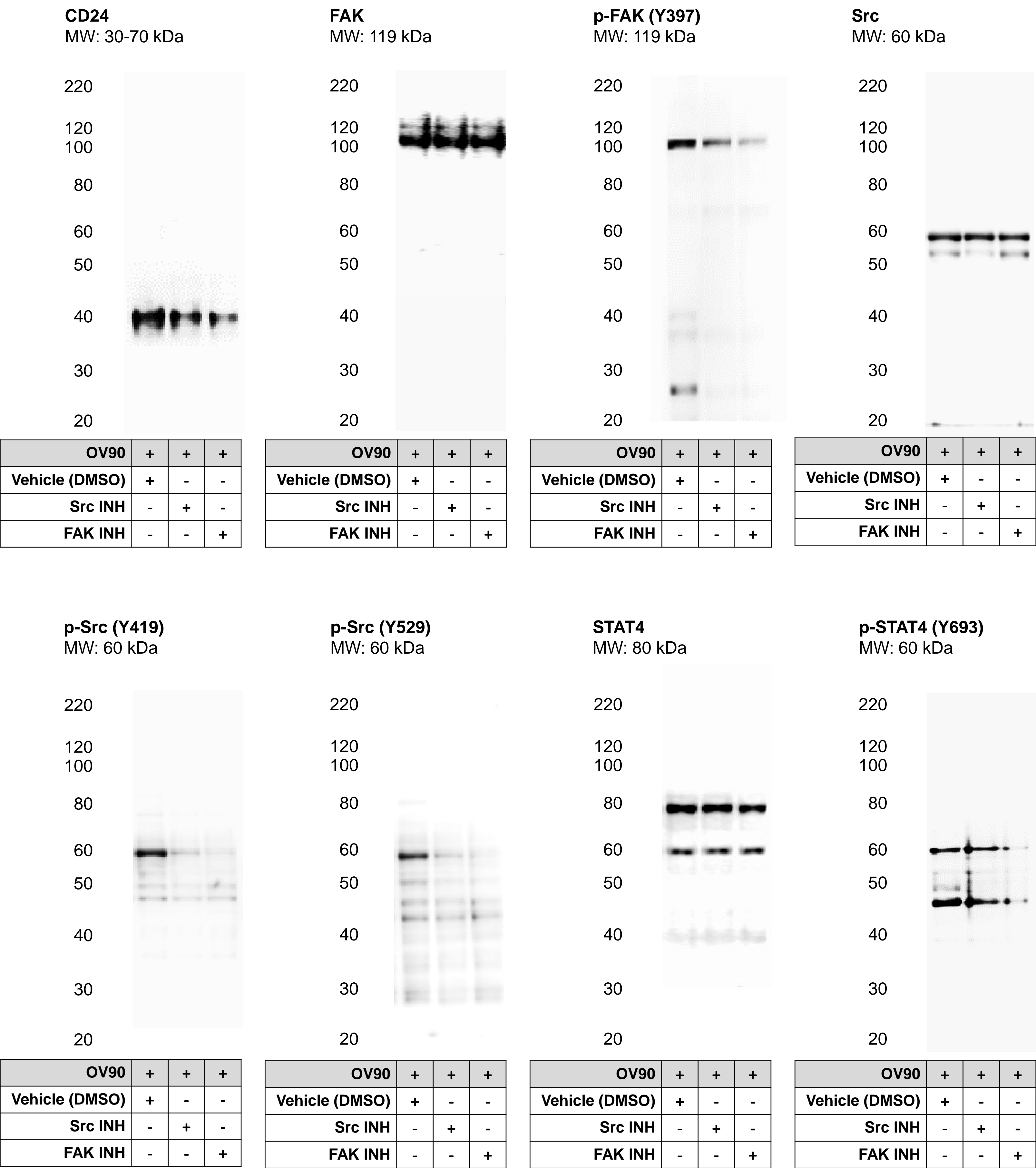

Figure 5G

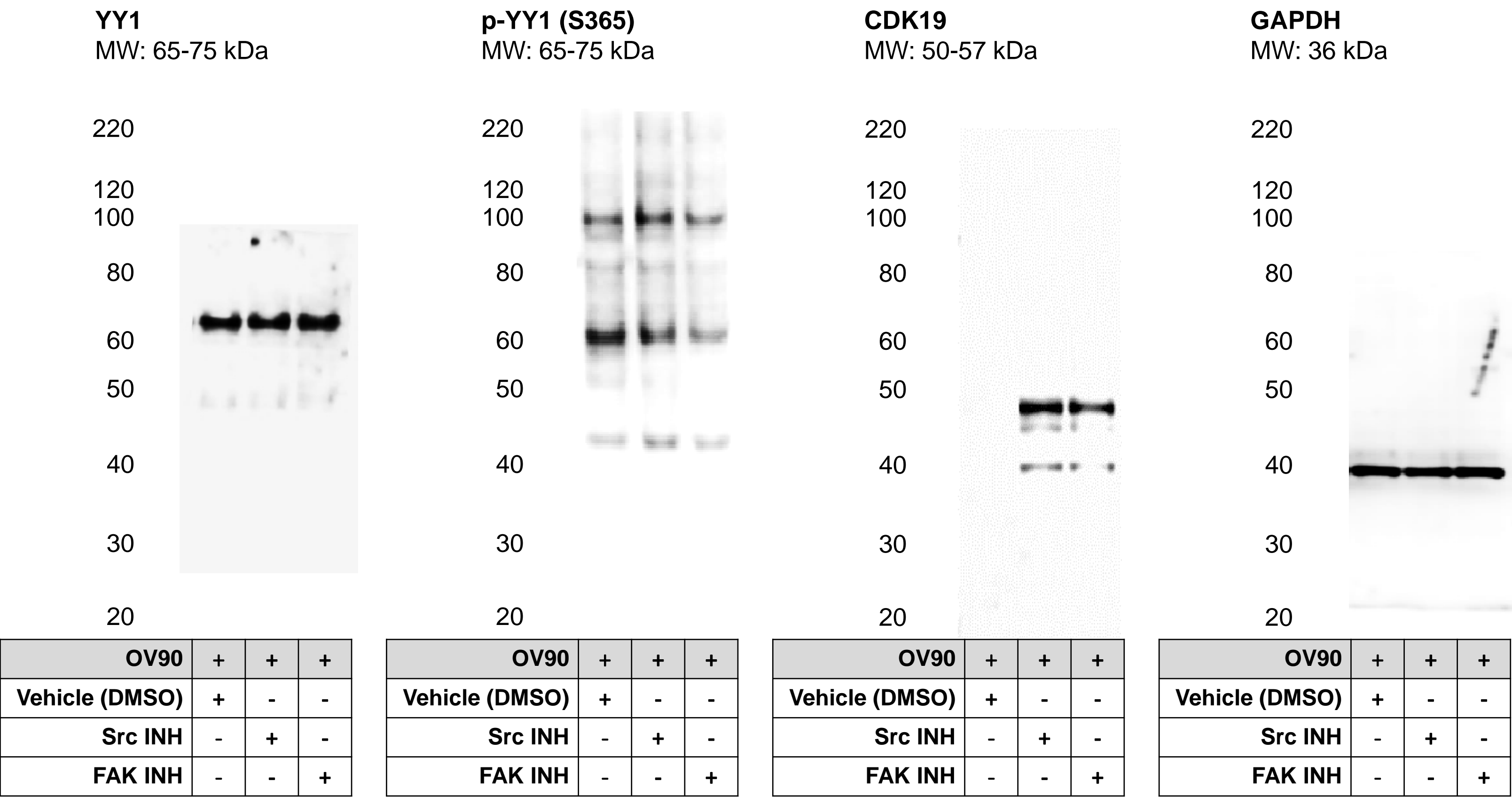

Supplement: Supplementary file 2 — Uncropped images of Western blot data [file 41420_2024_1858_MOESM2_ESM.pdf]
